# Intrastriatal injection of α-synuclein can lead to widespread synucleinopathy independent of neuroanatomic connectivity

**DOI:** 10.1186/s13024-017-0182-z

**Published:** 2017-05-29

**Authors:** Zachary A. Sorrentino, Mieu M.T. Brooks, Vincent Hudson, Nicola J. Rutherford, Todd E. Golde, Benoit I. Giasson, Paramita Chakrabarty

**Affiliations:** 1 Department of Neuroscience, University of Florida, 1275 Center Drive, PO Box 100159, Gainesville, FL 32610 USA; 2 Center for Translational Research in Neurodegenerative Disease, University of Florida, Gainesville, FL 32610 USA; 3 McKnight Brain Institute, University of Florida, Gainesville, FL 32610 USA; 4 Current address: Department of Neuroscience, Mayo Clinic, Jacksonville, FL -32224 USA

**Keywords:** α-synuclein, Amyloid, Prion, Striatum, Dopaminergic Neurodegeneration, Astrocyte

## Abstract

**Background:**

Prionoid transmission of α-synuclein (αSyn) aggregates along neuroanatomically connected projections is posited to underlie disease progression in α-synucleinopathies. Here, we specifically wanted to study whether this prionoid progression occurs via direct inter-neuronal transfer and, if so, would intrastriatal injection of αSyn aggregates lead to nigral degeneration.

**Methods:**

To test prionoid transmission of αSyn aggregates along the nigro-striatal pathway, we injected amyloidogenic αSyn aggregates into two different regions of the striatum of adult human wild type αSyn transgenic mice (Line M20) or non-transgenic (NTG) mice and aged for 4 months.

**Results:**

M20 mice injected in internal capsule (IC) or caudate putamen (CPu) regions of the striatum showed florid αSyn inclusion pathology distributed throughout the neuraxis, irrespective of anatomic connectivity. These αSyn inclusions were found in different cell types including neurons, astrocytes and even ependymal cells. On the other hand, intra-striatal injection of αSyn fibrils into NTG mice resulted in sparse αSyn pathology, mostly localized in the striatum and entorhinal cortex. Interestingly, NTG mice injected with preformed human αSyn fibrils showed no induction of αSyn inclusion pathology, suggesting the presence of a species barrier for αSyn fibrillar seeds. Modest levels of nigral dopaminergic (DA) neuronal loss was observed exclusively in substantia nigra (SN) of M20 cohorts injected in the IC, even in the absence of frank αSyn inclusions in DA neurons. None of the NTG mice or CPu-injected M20 mice showed DA neurodegeneration. Interestingly, the pattern and distribution of induced αSyn pathology corresponded with neuroinflammation especially in the SN of M20 cohorts. Hypermorphic reactive astrocytes laden with αSyn inclusions were abundantly present in the brains of M20 mice.

**Conclusions:**

Overall, our findings show that the pattern and extent of dissemination of αSyn pathology does not necessarily follow expected neuroanatomic connectivity. Further, the presence of intra-astrocytic αSyn pathology implies that glial cells participate in αSyn transmission and possibly have a role in non-cell autonomous disease modification.

**Electronic supplementary material:**

The online version of this article (doi:10.1186/s13024-017-0182-z) contains supplementary material, which is available to authorized users.

## Background

Intracellular accumulation of misfolded α-synuclein (αSyn) is a neuropathological hallmark of α-synucleinopathies [1–3]. Inclusions comprised of amyloidogenic αSyn aggregates, phosphorylated at Serine129 [4–6] are often found in neuronal cell bodies termed Lewy bodies (LB) or in neuronal projections, called Lewy neurites [1–3]. Nevertheless, how αSyn pathology initiates and spreads in the nervous system is still an area of intense research and debate.

In recent years, prion-like conformational templating has emerged as a plausible major mechanism that may explain the stereotypic progression of pathogenic αSyn into different brain regions [7–10]. Cell culture studies have shown that endogenous αSyn can be conformationally altered in the presence of exogenous pathologic αSyn to aggregate into inclusions [11–14], akin to classical prions [15, 16]. In vivo evidence for this prion-like characteristic of αSyn pathogenesis emerged from studies involving direct intracerebral injections of preformed αSyn amyloid fibrils in αSyn transgenic mice [17, 18]. More recently, studies have shown that peripheral (intramuscular, intraperitoneal or intravenous) injections of αSyn amyloid fibrils into rodents can induce progressive formation of αSyn pathology in the CNS [19–22]. However, the extent of induction and spread of seeded αSyn pathology in wild type nontransgenic (NTG) mice brains, on the other hand is more disparate; for example, while our group has observed that molecular templating and dissemination of pathogenic αSyn is an imprecise event in NTG mice [23], others have reported robust and widespread αSyn pathologies following exogenous αSyn challenge in NTG mice [14, 24–26]. Interestingly, injecting different molecular strains of αSyn can result in distinctive pathologies in rodents, possibly reflective of the spectrum of disease pathologies seen in different types of α-synucleinopathies [20, 27, 28]. Such dependence of seeded pathology on the presence of distinct conformer strains has been shown to exist in classical prion diseases [29]. Overall, these studies have established that αSyn can spread in the CNS by prion-like templating mechanisms [7–10].

Several recent publications have reported that following intracerebral injection of preformed αSyn fibrils, induction of intra-neuronal αSyn pathology leads to neurodegeneration, especially in the dopaminergic (DA) neurons of the nigrostriatal pathway [20, 24, 30]. This is reminiscent of selective neurodegeneration in α-synucleinopathies, such as Parkinson’s disease [31]. Understanding how intercellular transmission of pathological αSyn along specific neuroanatomic projections leads to selective neurodegeneration in Parkinsonisms and related Lewy body dementias will allow us to design better therapeutics against these diseases. To test whether intrastriatal injection of pathological αSyn aggregates leads to selective nigral degeneration and further whether this is dependent on overexpression of human αSyn transgene, we administered preformed αSyn aggregates into the internal capsule (IC) or the caudate putamen (CPu) areas of either Line M20 (transgenic mice expressing human wild type (WT) αSyn [32]) or NTG mice. Our results indicate that while induction of αSyn pathology was sparse in NTG mice, M20 mice showed extensive Lewy-type inclusion pathology widely distributed throughout the brain, irrespective of anatomic connectivity. Surprisingly, we did not observe any αSyn inclusion pathology in DA neurons and only modest levels of DA neurodegeneration were noticed in M20 mice injected in the IC. We observed induction of gliosis that is associated with incipient αSyn pathology and interestingly, massive levels of pathological αSyn inclusions were detected within astrocytes in M20 mice. Overall, our results suggest that a combination of glial and neuronal inclusion pathology may result in distinctive αSyn strains that display widespread patterns of dissipation throughout the CNS without obvious direct neuroanatomic connectivity.

## Methods

### Expression and purification of recombinant αSyn protein

The bacterial expression plasmid pRK172 cDNA construct encoding full-length WT human or mouse αSyn was used to express these proteins in *E. coli* BL21 (DE3) and they were purified to homogeneity by size exclusion (Superdex 200 gel filtration) followed by ion exchange (Mono Q) chromatographies as previously described [33].

### Fibril preparation of recombinant αSyn for mouse brain injection

Human αSyn recombinant protein or mouse αSyn recombinant protein was assembled into filaments by incubation at 37 °C at 5 mg/ml in sterile PBS (Invitrogen) with continuous shaking at 1050 rpm (Thermomixer R, Eppendorf, Westbury, NY). αSyn amyloid fibril assembly was monitored as previously described with K114 fluorometry [12]. αSyn fibrils were diluted to a concentration of 2 mg/mL in sterile PBS and gently sonicated at room temperature in a water bath sonicator for 1 h. This resulted in fragmentation of αSyn amyloid aggregates into shorter fibrils [17, 22]. These fibrils were imaged using transmission electron microscopy (EM) (Additional file 1: Figure S1), and were validated for induction of intracellular amyloid inclusion formation in cell culture as previously described [12]. For EM imaging, fibrils were adsorbed to 300-mesh carbon-coated copper grids, washed, stained with 1% uranyl acetate, and imaged at 100,000× magnification using a Hitachi H7600 transmission electron microscope (Hitachi).

### Mouse husbandry and stereotactic injections

**Fig. 1:**
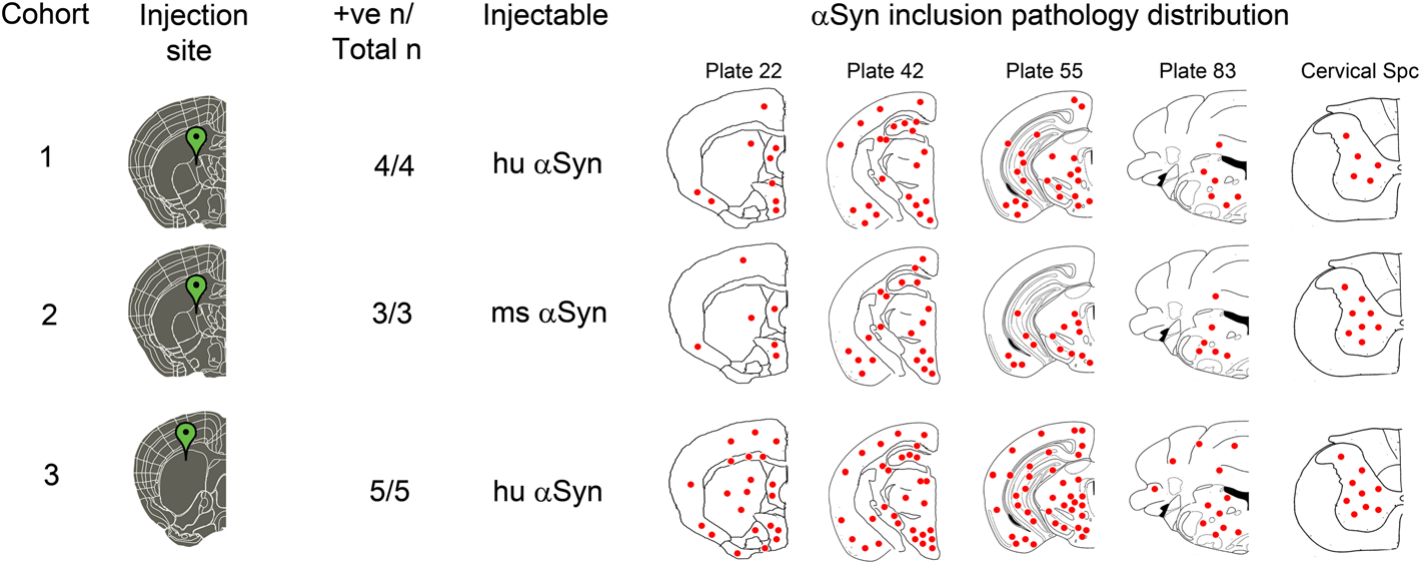
Summary of distribution of αSyn pathology in Line M20 mice following intrastriatal injection of human or mouse αSyn fibrils. In Cohorts 1 and 2, mice were injected with human (hu) WT αSyn fibrils (Cohort 1) or mouse (ms) WT αSyn fibrils (Cohort 2) in the IC area whereas Cohort 3 mice were injected with human WT αSyn fibrils in the CPu. The total number of mice injected in each cohort as well as the number of mice actually displaying αSyn pathology is depicted (denoted as +ve n/Total n). Rostro-caudal distribution of αSyn inclusions is presented on a schematic brain and spinal cord map, with red dots depicting the relative abundance of αSyn inclusions in different brain and spinal cord locations. The numbers on top of the brain map schematics correspond to plates from the Paxinos Brain Atlas [57]

All procedures were performed according to the NIH Guide for the Care and Use of Experimental Animals and were approved by the University of Florida Institutional Animal Care and Use Committee. Line M20 mice express human WT αSyn and do not develop any intrinsic phenotype or αSyn pathology [32]. 2 months old M20 or NTG mice were bilaterally injected with 2 μl of 2 mg/ml human or mouse WT αSyn fibrils in the IC (coordinates from Bregma: A/P -0.5, L +/−1.5, D/V -3.0; Cohorts 1, 2, 4 and 5) or CPu (coordinates from Bregma: A/P + 0.5, L +/−2.0, D/V -3.5; Cohort 3 and Cohort 6). The inoculum was injected at a rate of 0.2 μl per min with the needle in place for 15 min at each site. Mice were analyzed 4–5 months following injection. Cohort sizes are described in Figs. 1 and 3.

### Antibodies

The following antibodies were used to detect pathological αSyn phosphorylated at Ser129: 81A (B. Giasson; 1:10,000 for immunohistochemistry and 1:3000 for immunofluorescence; [5]) and EP1536Y (1:1000; AbCam, Cambridge, MA). The latter antibody specifically reacts with pSer129-αSyn and does not cross-react with phosphorylated low-molecular-mass neurofilament subunit [7]. Other antibodies used in this study are: Syn506, a conformation specific anti-αSyn antibody that preferentially detects αSyn in pathological inclusions (B. Giasson; 1:1000; [34]); anti-p62/sequestosome antibody (SQSTM1) (Proteintech, Chicago, IL); anti-glial fibrillary acidic protein (GFAP) (Dako, Carpentaria, CA); anti-Iba-1 antibody (1:1000; Wako, Richmond, VA) and anti-tyrosine hydroxylase (TH) antibody (Millipore, Billerica, MA).

### Immunohistochemical and immunofluorescence analyses

Mouse brains and spinal cords were collected following intra-cardiac perfusion with PBS/heparin. Tissues were fixed with 70% ethanol/150 mM NaCl for 48 h followed by paraffin processing. Paraffin-embedded tissue sections were deparaffinized and hydrated through a series of graded ethanol solutions followed by washing with 0.1 M Tris, pH 7.6. The sections were blocked with 2% FBS in 0.1 M Tris, pH 7.6. Immunohistochemical detection was done using avidin-biotin complex (ABC) system (Vectastain ABC Elite Kit, Vector Laboratories, Burlingame, CA) and immunocomplexes were visualized with the chromogen 3,3′-diaminobenzidine (DAB). Sections were counterstained with hematoxylin. Slides were scanned using an Aperio ScanScope CS (Aperio Technologies Inc., Vista, CA) and images acquired using the ImageScopeTM software (Aperio Technologies Inc.). Quantification of immunostaining was done using the Pixel count Program (ImageScope, Aperio Technologies). For immunofluorescence detection, sections were incubated with secondary antibodies conjugated to Alexa fluor 594 or Alexa fluor 488 (Invitrogen, Eugene, OR) followed by Sudan Black treatment and staining with DAPI (Invitrogen, Eugene, OR). The sections were coverslipped with Fluoromount-G (Southern Biotech, Birmingham, AL) and visualized using an Olympus BX51 microscope mounted with a DP71 Olympus digital camera.

### Stereological analysis of TH neurons in the substantia nigra

TH-positive neurons in the substantia nigra (SN) were counted by staining paraffin-embedded sections with an anti-TH polyclonal antibody (Millipore, Billerica, MA). Stereological counting of DA neurons was performed by counting and tallying TH-immunopositive neurons on every tenth section based on a previously published report [35].

## Results

### Induction and spread of pathological αSyn in M20 mice does not strictly follow neuroanatomic projections

**Fig. 2:**
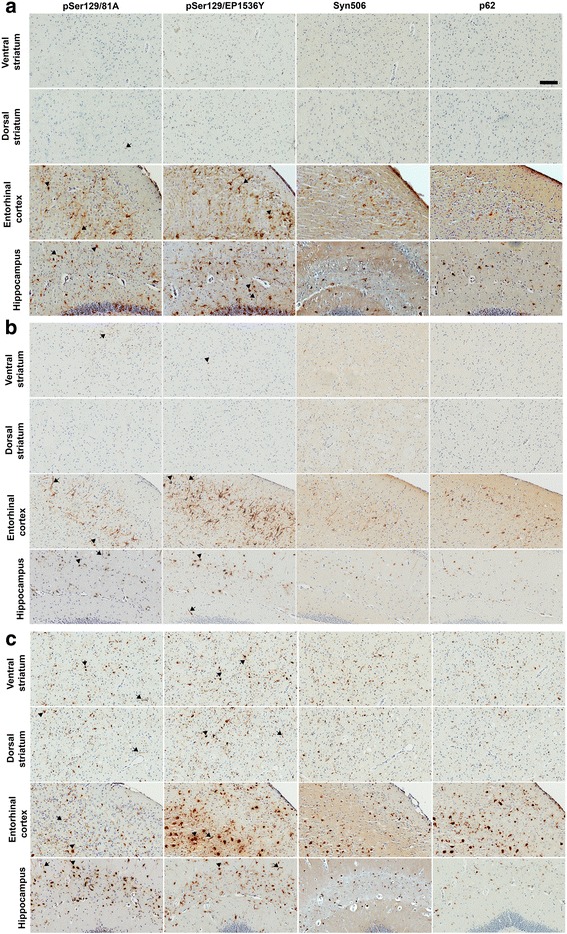
Induction of αSyn pathology following injection of αSyn fibrils in the IC of line M20 mice. 2 month old Line M20 mice were injected with pre-formed human WT αSyn fibrils in IC (**a**, Cohort 1) or mouse WT αSyn fibrils in IC (**b**, Cohort 2) or human WT αSyn fibrils in the CPu (**c**, Cohort 3) and analyzed after 4 months. Abundant αSyn inclusion pathology, detected by pSer129-αSyn antibodies (81A and EP1536Y) was observed in most areas of the brain, even those that are not directly connected to the striatum, such as the hippocampus. Surprisingly, areas adjacent to the IC, such as the ventral and dorsal striatum had few pathological inclusions. Inclusion pathology was confirmed using a conformation-specific antibody against αSyn, Syn506, and p62/Sqstm1 antibody, which is a well-established marker of cytoplasmic LB inclusions. Both perikaryal (*arrowhead*) and neuritic (*arrow*) αSyn pathology were observed. The relative distribution and abundance of αSyn pathology in Cohort 3 was similar to Cohort 1, except that mice in Cohort 3 had relatively higher density of αSyn inclusion pathology in the striatum. Note that since 1 mouse in Cohort 2 died prematurely from hindlimb paralysis, neuropathological analysis could not be performed. Scale Bar, 100 μm; *n* = 3–5 mice

To test the idea that induction of αSyn pathology follows pre-determined neuroanatomic pathways, we injected aggregated WT αSyn fibrils in two distinct regions of the striatum of 2 month old line M20 mice that overexpress human WT αSyn [32] as well as their NTG littermates. The M20 mice never intrinsically develop αSyn inclusion pathology or have overt phenotypes [32]. These injections were done to enable us to (1) study the efficiency of templated induction of WT αSyn pathology and (2) further conduct studies in NTG mice that are much less inherently susceptible to induction of αSyn inclusion pathology [23] so that we could better visualize and interpret frank transmission occurring via neuroanatomic connectivity. Cohorts 1 and 2 of M20 mice received bilateral injections into the IC while the Cohort 3 received bilateral injections in the CPu (Fig. 1). The IC, containing myelinated corticospinal tracts, intersects the caudate nucleus and putamen of the striatum. It serves as a conduit between motor areas, frontopontine, and thalamic peduncles to brain stem and cerebellar regions, and also between the thalamus and prefrontal cortex. Examination of Cohort 1 injected with human αSyn fibrils (Figs. 1 and 2a) or Cohort 2 injected with mouse αSyn fibrils (Figs. 1 and 2b) showed similar distribution of pSer129 αSyn/81A–immunopositive pathologies. αSyn pathology was further confirmed by staining sections with an additional antibody against pSer129-αSyn (EP1536Y) as well as an antibody raised against conformationally altered αSyn (Syn506). Immunostaining with p62/sequestosome confirmed that the intraneuronal and neuritic αSyn staining represented frank inclusion pathology (Fig. 2a-b). In both cohorts, robust αSyn inclusion pathology was found spread over multiple neuroanatomically connected areas as well as other areas of the brain that have no known direct connections to the IC. Overall, human αSyn fibril injected M20 mice (Cohort 1) showed profuse αSyn pathology in motor cortex, piriform cortex, somatosensory cortex, amygdala (basolateral and basomedial nuclei), ventral thalamus, IC, hypothalamus (ventromedial and dorsomedial), hippocampus, dentate gyrus, retrosplenial cortex, geniculate nucleus, SN region, retromammillary body, posterior commissure, cerebellar folia, vestibular nuclei and spinal cord (Figs. 1 and 2a, Additional file 2: Figure S2). Surprisingly, we observed very limited αSyn pathology in the CPu region. In M20 mice injected with mouse αSyn fibrils (Cohort 2), αSyn pathology was observed in all of these areas except there was sparse to no pathology in somatosensory cortex and retrosplenial cortex (Figs. 1 and 2b, Additional file 2: Figure S2). On the whole, this data shows that the induction and subsequent dissemination of the template αSyn pathology in M20 mice is widespread and not strictly restricted to areas that are neuroanatomically connected to the IC.

Since the IC provides connectivity between various brain areas supporting multiple, diverse, and dissociable functional regions, we next investigated whether injecting human αSyn fibrils directly into the CPu resulted in transmission of induced αSyn pathology into discrete areas linked by direct projections from this area (Cohort 3). The CPu area receives inputs from the cortex and sends projections directly into the globus pallidus, thalamus, and SN. Mice were examined for αSyn inclusion pathology following injection of human αSyn fibrils into the CPu. These M20 mice showed essentially a similar distribution of αSyn pathology as observed in Cohort 1 and Cohort 2 mice (Figs. 1 and 2c), indicating that templated induction of αSyn pathology follows similar patterns of distribution whether initiated in a white matter enriched area (IC) or a projection rich area (CPu). All M20 cohorts (Cohorts 1, 2 and 3) showed extensive perikaryal as well as axonal pathology; additionally, Cohort 3 mice showed pathology throughout the cortex especially in the visual and entorhinal cortex (Figs. 1 and 2c, Additional file 2: S2). On the whole, Cohort 3 of M20 mice showed relatively more robust and widespread distribution of αSyn inclusion pathology than mice injected with human αSyn fibrils or mouse αSyn fibrils in the IC (Cohorts 1, 2) (Fig. 2a-c). An important distinction between these cohorts is that the Cohort 3 mice seemed to develop relatively more robust αSyn pathology in and around the area of injection, i.e., striatum, than Cohort 1 and 2 mice, which had limited αSyn pathology proximal to the area of injection. We also observed robust induction of αSyn inclusion pathology in the ependymal cells abutting the lateral ventricles in the human αSyn fibril injected M20 mice in Cohorts 1 and 3 (Additional file 3: Figure S3).

### Induction and spread of pathological αSyn in NTG mice is sparse and restricted

**Fig. 3:**
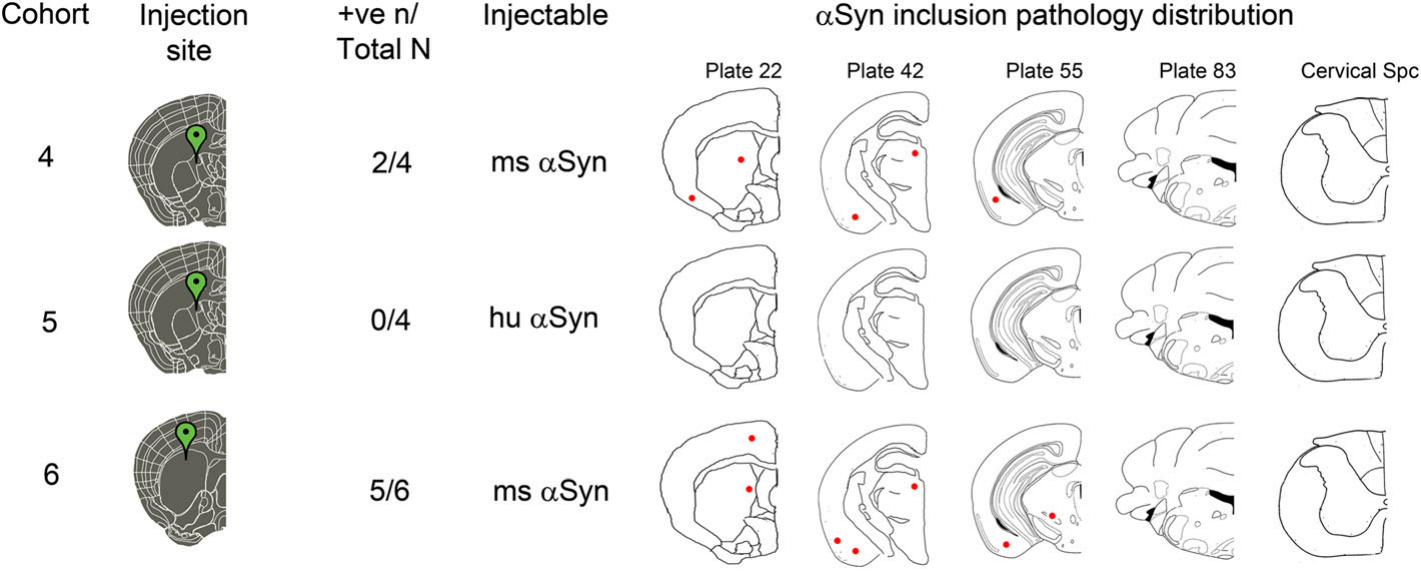
Summary of distribution of αSyn pathology in NTG mice following intrastriatal injection of human or mouse αSyn fibrils. NTG mice were injected with mouse WT αSyn fibrils (Cohort 4) or human WT αSyn fibrils (Cohort 5) in the IC area whereas Cohort 6 mice were injected with mouse WT αSyn fibrils into the CPu. The total number of mice in each cohort as well as the number of mice displaying induced αSyn pathology is shown (denoted as +ve n/Total n). Rostro-caudal distribution of inclusions is presented on schematic brain maps, with red dots depicting the relative abundance of αSyn inclusions in different brain and spinal cord locations. The numbers on top of the brain schematic correspond to plates from the Paxinos Brain Atlas [57]

**Fig. 4:**
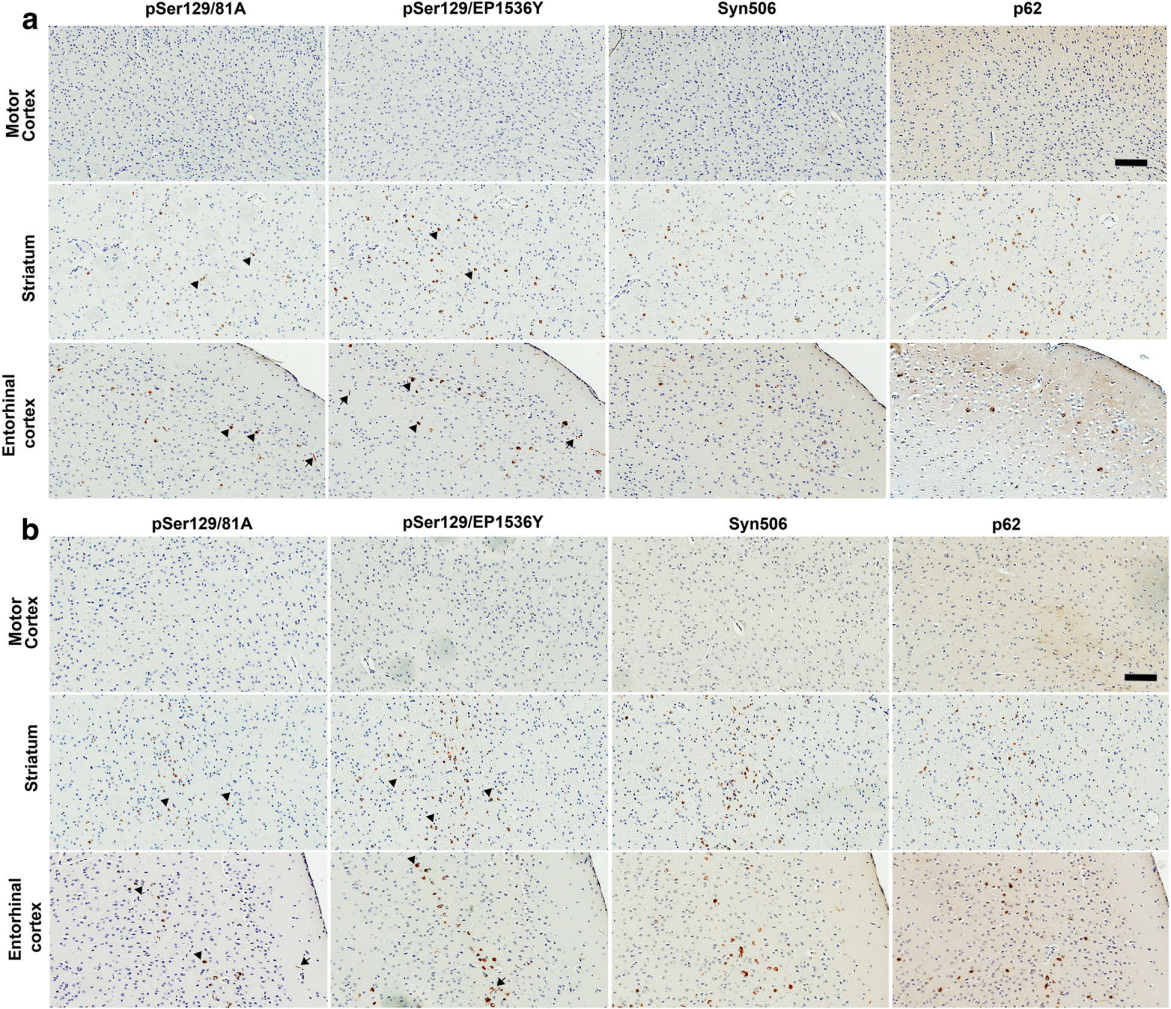
Limited induction of αSyn pathology following injection of mouse αSyn fibrils in NTG mice. 2 month old NTG mice were injected in the IC with mouse WT αSyn fibrils (**a**, Cohort 4) or injected in the CPu with mouse WT αSyn fibrils (**b**, Cohort 6) and analyzed after 4 months. Immunostaining with pSer129-αSyn specific antibodies (81A and EP1536Y), conformation-specific αSyn antibody (Syn506), and p62/Sqstm1 antibody shows induction of αSyn inclusion pathology limited exclusively to the striatum and entorhinal cortex. In Cohort 4, only 2 mice (total *n* = 4) showed appreciable αSyn pathology. Most of the αSyn pathology was restricted to perikaryal LB-like inclusion pathology in these 2 mice (*arrow heads*). Both cohorts showed extremely limited induction of αSyn pathology. No αSyn pathology was observed in the motor cortex (top panels, **a** and **b**). Scale Bar, 100 μm; *n* = 4–6 mice

In our previous studies, we found that following intrahippocampal seeding, the templated induction of αSyn pathology in NTG mice is a relatively inefficient process compared to αSyn transgenic M20 mice [17, 23]. We reasoned that if there was any frank propagation of pathology along neuroanatomic connections, we would be able to clearly visualize this phenomenon in NTG mice that otherwise do not develop a surfeit of αSyn pathology outside of the area of injection. To characterize propagation of conformationally altered αSyn in NTG mice, we injected mouse αSyn fibrils (Cohort 4) or human αSyn fibrils (Cohort 5) into the IC (Figs. 3 and 4a) or mouse αSyn fibrils in the CPu (Cohort 6) (Figs. 3 and 4b). Cohort 5 had no detectable αSyn pathology and therefore no further analysis was done in these mice (data not shown). We noticed that in Cohorts 4 and 6, αSyn inclusion pathology was observed in dorsal striatum (CPu), thalamus, piriform cortex and entorhinal cortex (Figs. 3 and 4). Mice injected in the IC did not show appreciable αSyn pathology in the motor cortex, which is unexpected given that the IC is richly populated by corticospinal tracts (Figs. 3 and 4b). In both of these cohorts of NTG mice, the majority of the αSyn inclusions were perikaryal, with limited neuritic pathology (Fig. 4). Neither of these two cohorts showed any appreciable αSyn pathology in other areas that are neuroanatomically connected to the striatum, e.g., thalamus and SN region. Additionally, we did not observe any αSyn pathology in ependymal cells (Additional file 3: Figure S3), cerebellum or spinal cord (data not shown) of NTG mice.

### Intra-striatal administration of αSyn aggregates does not necessarily induce degeneration of dopaminergic neurons in the substantia nigra

**Fig. 5:**
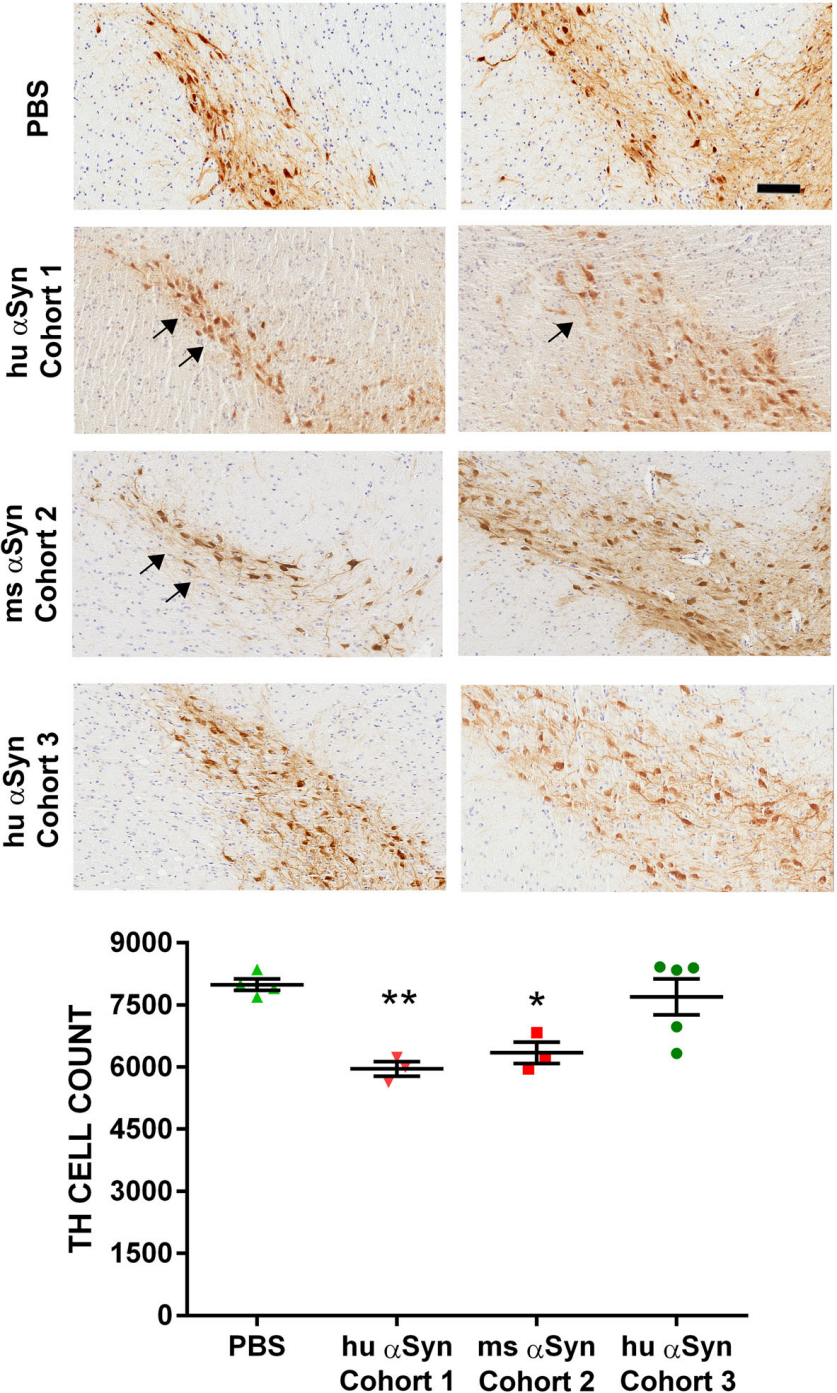
Modest levels of nigral DA neuronal loss in line M20 mice injected in IC with αSyn fibrils. Line M20 mice were injected in the IC (Cohorts 1 and 2) or CPu (Cohort 3) with WT αSyn fibrils. Representative TH staining depicting SN-resident DA neurons in the dorsal (left panel) and ventral aspects (right panel) are shown. Mice that were injected with either human αSyn fibrils (Cohort 1) or mouse αSyn fibrils (Cohort 2) showed modest levels of DA degeneration as determined by counting TH-immunostained neurons in the SN (~20–25% compared to PBS injected control mice). The thinning of the nigral TH neuron layer is indicated by *arrows*. Cohort 3 mice injected with human αSyn fibrils did not display any nigral degeneration. PBS injected Line M20 mice were considered as control group for all cohorts. Scale Bar, 100 μm; *n* = 3–5/cohort. One way Anova, **p < 0.01, *p < 0.5

**Fig. 6:**
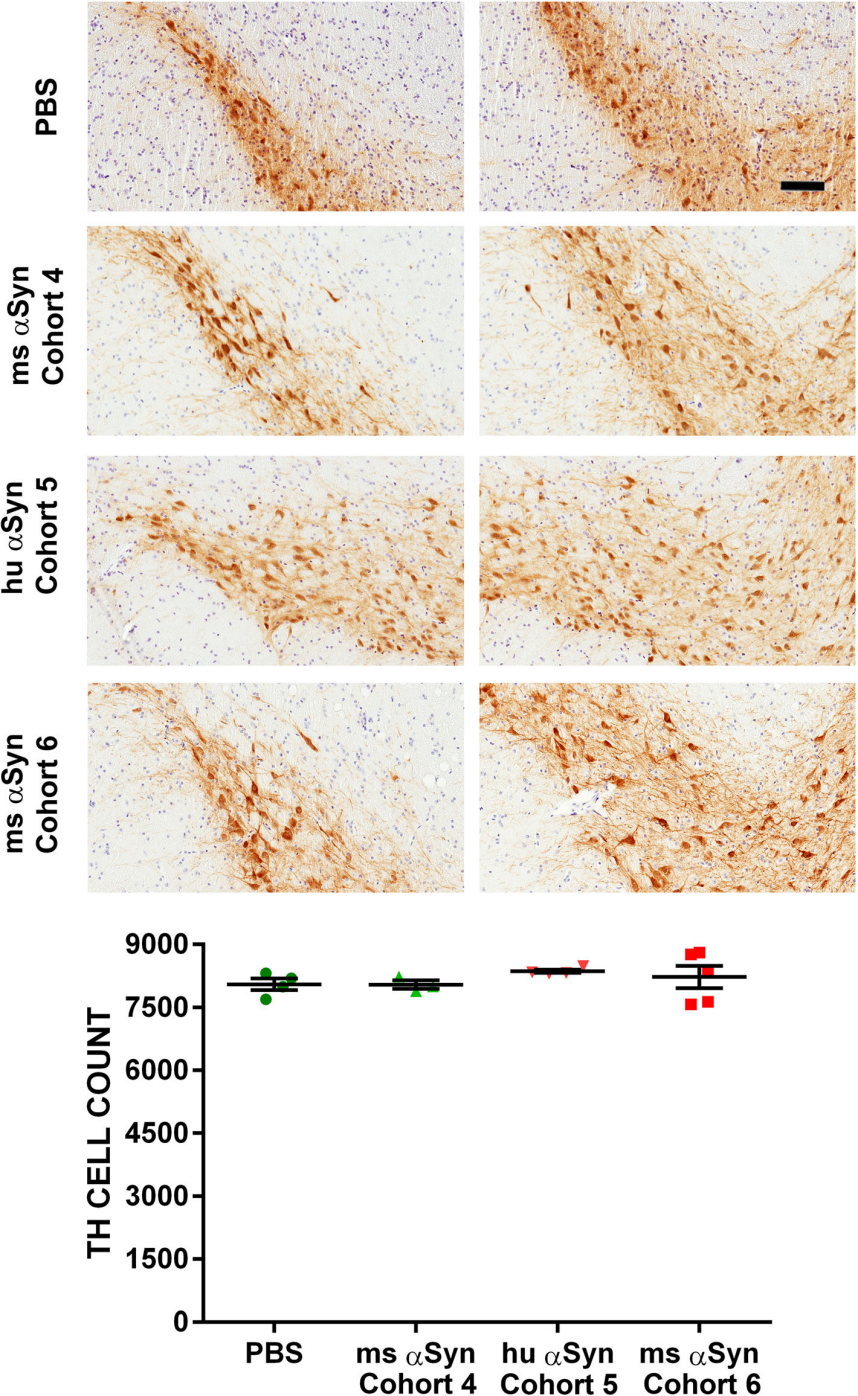
Intrastriatal injection of αSyn fibrils in NTG mice does not result in nigral DA neurodegeneration. NTG mice were injected in the IC (Cohort 4, injected with mouse αSyn fibrils and Cohort 5, injected with human αSyn fibrils) or CPu (Cohort 6; injected with mouse αSyn fibrils). Representative TH staining depicting DA neurons in the dorsal and ventral aspects of SN are shown (left and right panels respectively). The total number of TH-immunostained neurons were unaltered in all cohorts compared to PBS injected mice. Scale Bar, 100 μm; *n* = 3–5/cohort

Tractographic studies have conclusively established that striatal neurons (both dorsal and ventral) project monosynaptically to DA neurons in the SN [36]. Especially, a majority of projections from the posterior aspect of the striatum has been shown to lead into the DA neurons of SN and retrorubral nucleus [37]. Therefore, we expected that direct injections of αSyn fibrils into the dorsal striatum (CPu) or IC (situated between caudate and putamen) should result in targeting DA neurons in the SN. Though we observed pSer129 αSyn immunopositive inclusions in the SN region and striatum of M20 transgenic mice, the αSyn inclusion pathology did not co-localize with the TH-immunopositive DA neuronal cell bodies (Additional file 4: Figure S4). We then conducted stereologic counting of TH-immunopositive DA neurons in M20 (Cohorts 1–3) or NTG mice (Cohorts 4–6) to explore whether seeded αSyn pathology affects survival of DA neurons in the SN. We observed a modest reduction in number of DA neurons in M20 transgenic mice injected in the IC with human αSyn fibrils (↓25.5%; Cohort 1) or mouse αSyn fibrils (↓20.6%; Cohort 2) (Fig. 5). Surprisingly, M20 transgenic mice injected with human αSyn fibrils in the CPu (Cohort 3) did not show any DA neuronal loss (Fig. 5). None of the NTG mice injected with either human αSyn fibrils (Cohort 5) or mouse αSyn fibrils (Cohorts 4 and 6) showed any nigral neuronal loss (Fig. 6).

### Characterization of astrocytosis and microgliosis in αSyn seeded mice

**Fig. 7:**
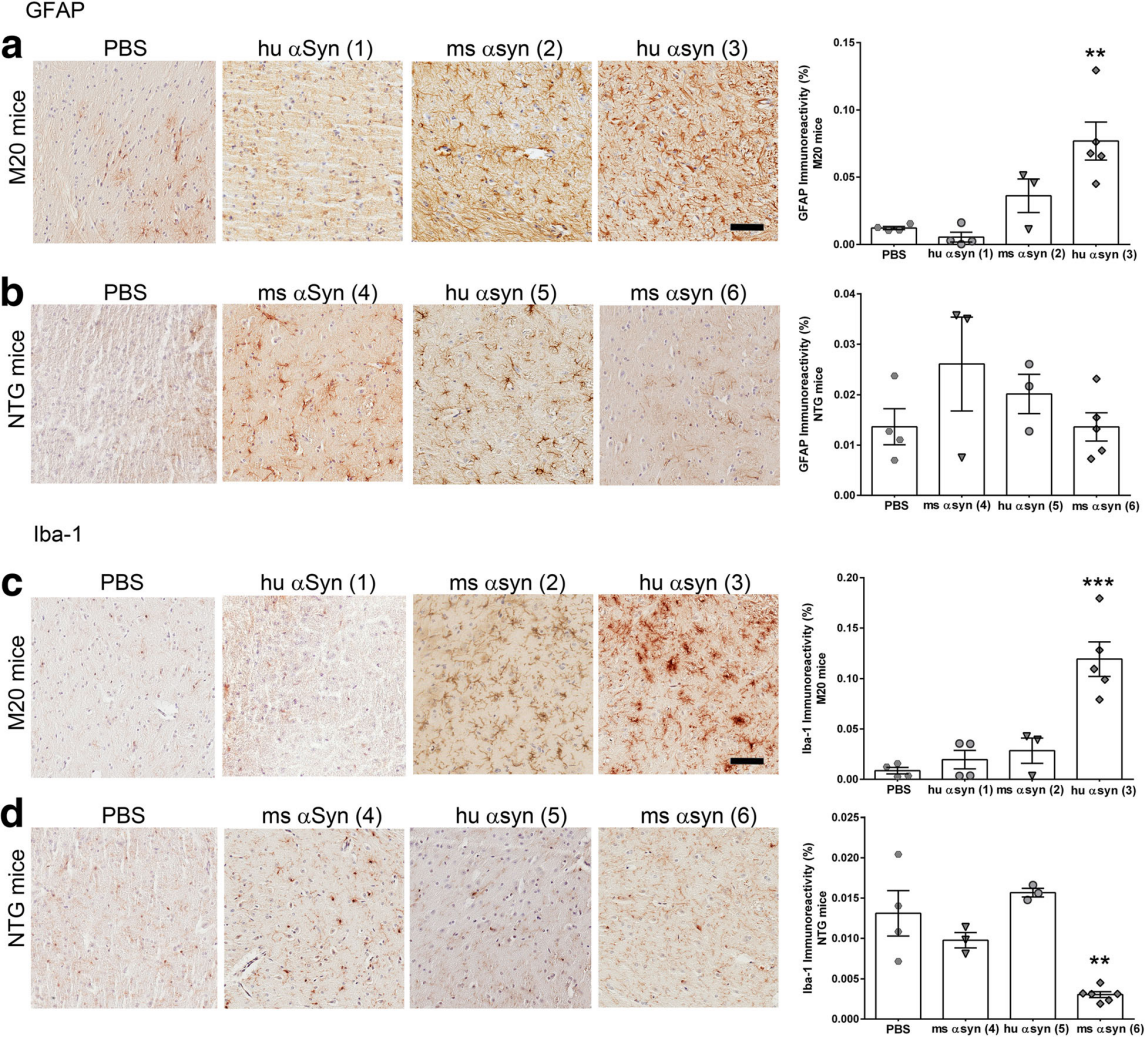
Increased astrogliosis in line M20 mice injected with αSyn fibrils. Representative images of astrocytosis (GFAP immunostaining, **a**-**b**) and microgliosis (Iba-1 immunostaining, **c**-**d**) in the striatum region of line M20 (**a**, **c**) or NTG (**b**, **d**) mice injected with αSyn fibrils. Astrocytic and microglial activation was significantly upregulated in line M20 mice injected in the CPu with human αSyn fibrils (Cohort 3). Astrogliosis was variable in Cohort 1 of line M20 mice whereas in Cohort 2 of M20 mice, injection of mouse αSyn fibrils in the IC resulted in an insignificant trend towards increased GFAP and Iba-1 reactive astroglia. In all cohorts of NTG mice, the extent of astrogliosis was more variable (Cohorts 4, 5 and 6) and in Cohort 6, mice showed significantly lower microgliosis compared to control group. In all cases, genotype-matched PBS injected mice served as controls. Different cohorts are identified by numbers in parenthesis as described in Figs. 1 and 3. Scale Bar, 100 μm; *n* = 3–5/cohort; 1 way Anova, ***p < 0.005, **p < 0.01

To understand how gliosis is altered in αSyn seeded milieus, we conducted region-wise analysis of astrocytes and microglia in M20 and NTG mice. Overall, the induction of astrogliosis was much more consistently reproducible in M20 mice injected in the CPu (Cohort 3) compared to all other cohorts. In M20 mice, injection in CPu with human αSyn fibrils (Cohort 3) resulted in the most striking and consistent increases in astrocytosis (GFAP immunostaining) (Fig. 7a) and microgliosis (Iba-1 immunostaining) (Fig. 7c) in the striatum. Other areas distal to the injection sites that displayed robust αSyn deposits in these mice, such as the hippocampus and entorhinal cortex, also showed increased GFAP and Iba-1 staining, albeit with more individual variability compared to striatum (Additional file 5: Figure S5, Fig. 7a and c **)**.

Line M20 mice injected in IC (Cohorts 1 and 2) showed much more variability in astrogliosis across individual mice, in spite of consistently uniform distribution of αSyn pathology in these mice. In particular, Cohort 1 mice showed no induction of gliosis in and around the area of injection (i.e., striatum) (Fig. 7a), whereas there was increased astrogliosis and microgliosis in hippocampus and entorhinal cortex, albeit with more mouse-to-mouse variability; therefore, in a facile sense, induction of astrogliosis cannot be directly explained by the accretion of induced αSyn pathology (Additional file 5: Figure S5). Interestingly, there is also discordance between GFAP and Iba-1 immunostaining that is strikingly evident in the M20 mice injected in the IC (Cohorts 1 and 2) – for example, there was generally higher levels of astrogliosis in Cohort 2 compared to limited alterations in microgliosis (Fig. 7a and c; Additional file 5: Figure S5).

The extent of astrogliosis and microgliosis in NTG mice was variable throughout brain regions in the αSyn injected cohorts. Overall, in NTG mice injected in the IC (Cohorts 4 and 5) or CPu (Cohort 6), we observed sparse astrogliosis (Fig. 7b and d, Additional file 6: Figure S6). For example, Cohort 5 of NTG mice with no induction of αSyn inclusion pathology (i.e., injected in IC with human αSyn fibrils) expectedly does not show increased astrogliosis. Interestingly, with respect to mouse αSyn fibril injected cohorts, mice injected in the white matter rich IC area (Cohort 4) display higher (but variable) astrocytosis than in mice injected in the CPu (Cohort 6) (Fig. 7b, Additional file 6: Figure S6). Surprisingly, in spite of the presence of αSyn pathology, microgliosis was significantly downregulated in the striatum, hippocampus and entorhinal cortex of NTG mice injected with ms αSyn fibrils in the CPu (Cohort 6; Fig. 7d, Additional file 6: Figure S6).

### Induction of robust αSyn pathology within nigral astrocytes but not TH neurons in M20 mice

**Fig. 8:**
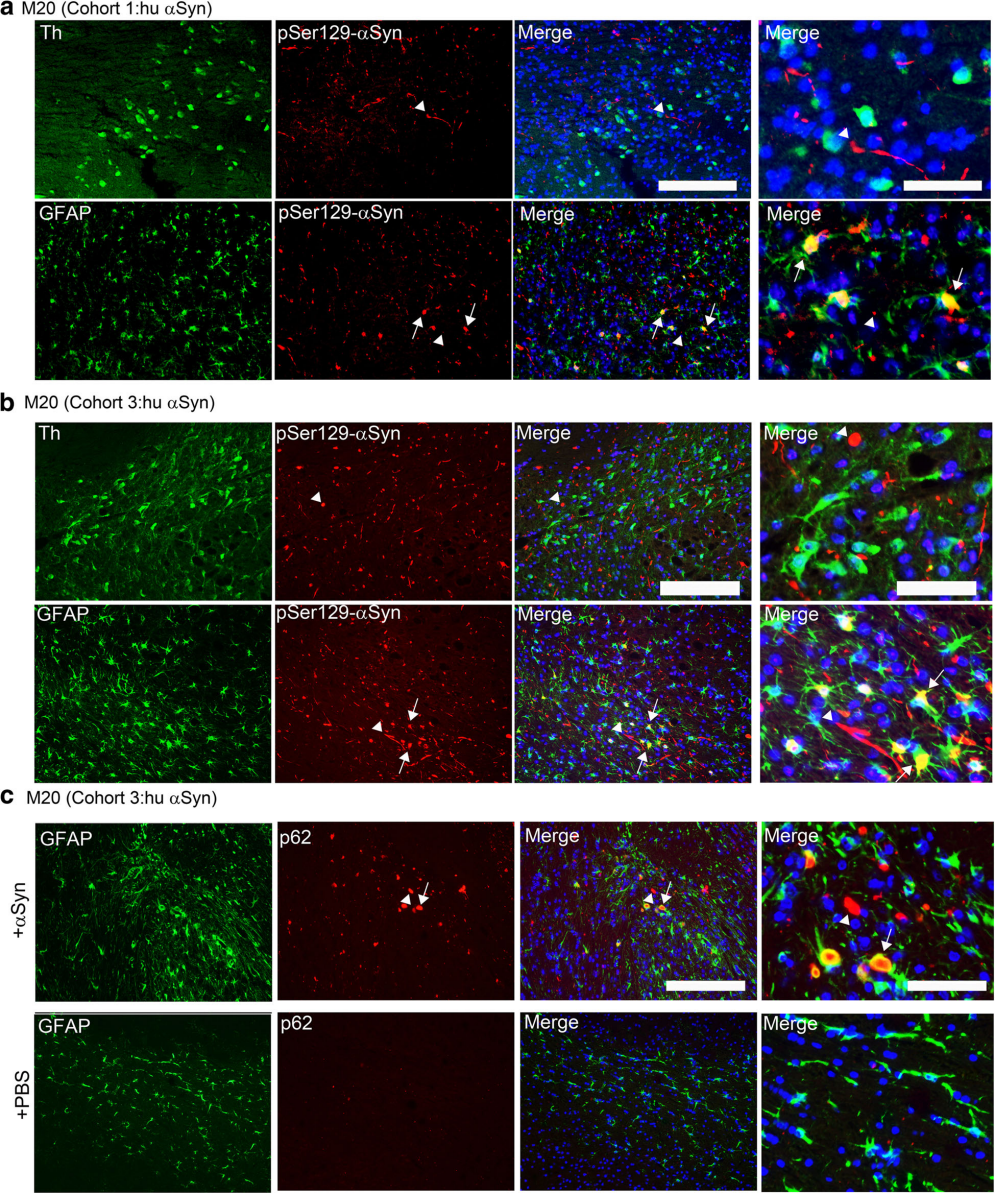
αSyn aggregates preferentially co-localized within astrocytes but not in SN-resident DA neurons in line M20 mice injected with human WT αSyn fibrils. Representative immunofluorescence staining showing co-localization of pSer129 immunoreactive αSyn pathology (*red*) in the TH immunopositive DA neurons (*green*) or GFAP (*green*) immunopositive astrocytes in the SN region of human αSyn fibril injected M20 mice. In both cohorts of M20 mice injected in the IC or the CPu (**a**-**b**: Cohorts 1 and 3 respectively), astrocytes laden with pSer129-αSyn were present in abundance (*arrows*) while none of these LB type inclusions were identified within the TH-immunopositive DA neurons. Additionally, many cell body inclusions or neurites were present in populations that were not immunopositive for TH (*arrowheads*). Of note, the astrocytes with resident αSyn pathology resemble hypermorphic reactive state and co-localized with p62, an indicator of decreased autophagic flux (**c**). PBS injected mice represents the control mice (**c**). Cell nuclei were stained with DAPI (*blue*). The 3-color merged panel has been magnified (right panel) to visualize the localization of astrocytic pSer129 αSyn pathology in the SN. *n* = 3–5/cohort; Scale bar, 500 μm (all panels except right panel); 200 μm (merged panel, right)

In α-synucleinopathies, glial αSyn pathology is posited to contribute to progression of the disease and may be directly associated with DA neurodegeneration [38]. Therefore, we next investigated the localization of pSer129-αSyn pathology in glial cells and TH-immunopositive nigral DA neurons in M20 mice that showed frank nigral neurodegeneration (Cohort 1) (Fig. 8a) compared to M20 mice with no DA degeneration (Cohort 3) (Fig. 8b). In both these cohorts, which had widespread αSyn pathology and astrocytosis but differential DA neurodegeneration in the SN, we rarely observed any TH-resident pathological αSyn inclusions (Fig. 8a-b, Additional file 4: Figure S4B-C). Interestingly, a copious amount of pSer129-αSyn pathology was resident within the GFAP-immunopositive astrocytes in the SN in both these cohorts (Fig. 8a-b). We next tested whether astrocytic inclusion pathology was associated with autophagic alterations. Since p62 accumulates when autophagy is inhibited, and decreased levels can be observed when autophagy is induced, p62 may be used as a marker to study autophagic flux [39]. We noticed that these astrocytic inclusions were indeed immunopositive for p62, a cellular marker indicating inhibition of autophagy (Fig. 8c).

In Cohort 3, astrocyte resident pSer129-αSyn pathology was also observed in several other brain areas that had induced αSyn pathology, such as ventral midbrain, hippocampus and cortex in these mice (Additional file 7: Figure S7b). We also observed a similar distribution of astrocytic pSer129-αSyn pathology in Cohort 1 of M20 mice injected with human αSyn fibrils, though individual mice in this cohort showed variable induction of astrogliosis (Additional file 7: Figure S7A). In the NTG cohorts displaying induced αSyn pathology (Cohorts 4 and 6), we did not observe any astrocytic αSyn pathology in the ventral midbrain, entorhinal cortex or hippocampus (Additional file 8: Figure S8A–B).

## Discussion

Templated conformational alterations in αSyn has been shown to induce LB-like pathology in mice, suggesting that this maybe a significant disease mechanism in human α-synucleinopathies [7–10]. While some reports have indicated that induced αSyn pathology spreads along neuroanatomic connections in a prionoid fashion [18, 20, 24–26, 40], we and others have previously reported that propagation of seeded αSyn pathology in mice can follow atypical or non-prionoid characteristics [17, 21, 41, 42]. Here, using human WT αSyn transgenic mice (M20) and NTG mice, we show that 1) injection of αSyn aggregates into two different areas of striatum results in widespread αSyn inclusion pathology in M20 mice, irrespective of neuroanatomic connectivity; 2) templated αSyn pathology following intra-striatal injection is not transmitted to DA neurons and causes moderate to no degeneration in DA neurons; 3) induction of αSyn pathology can be an inefficient process and spares the SN-resident TH-immunopositive neurons in NTG mice; and 4) αSyn laden astrocytes were readily observed in brain regions of M20 mice with robust αSyn pathology, suggesting that non-neuronal cells can also contribute to the dissemination of αSyn pathology. Overall, our observations indicate that, in addition to inter-neuronal transfer of seeded αSyn pathology along anatomic connections, simple diffusion or glia-mediated regional transfer may also affect the extent and distribution of pathologies in mouse models of α-synucleinopathies. In our study, a significant proportion of αSyn pathological inclusions induced by the administration of exogenous αSyn fibrils were localized within astrocytes throughout the CNS, in stark comparison to other published studies that have shown minimal glial involvement [18, 26]. It is possible that this unique property of our αSyn preparations along with potential maturation of αSyn pathology within the astroglia may generate unique αSyn conformers with distinctive strain-like properties that are preferentially transmitted via glial cells relative to direct inter-neuronal transfer. Induction of α-synucleinopathy by such astrocyte-discriminative αSyn conformers could be used to mechanistically understand the disease etiology in α-synucleinopathies with massive glial involvement, such as in multiple system atrophy [43, 44]. Whether the αSyn fibrils used in our studies indeed possess a preference for enhanced glial transmission relevant to disease pathogenesis in distinct spectra of α-synucleinopathies and how this impacts the progression and outcome of different subtypes of α-synucleinopathies will be examined in future studies.

### Critical parameters that govern dissemination of αSyn seeds: concept of endogenous barriers

In the prion field, permissive transmission depends on overcoming the species barrier, which is determined by a range of possible conformers of a particular prion, its sequence, as well as its interaction with cellular co-factors such as chaperones [45–47]. One such endogenous ‘barrier’ relevant to α-synucleinopathies may be the subtle differences in the sequence of mouse and human αSyn (7/140 amino acids difference) [48] or their differential interactions with cellular co-factors. Based on this premise, mouse and human αSyn may have differential abilities in recruiting endogenous αSyn into pathological aggregates [26]. In support of this theory, we observed that in NTG mice, while mouse αSyn aggregates resulted in induction of αSyn pathology, human αSyn fibrils were ineffective. Therefore, it is likely that a ‘species barrier’ may determine the structural templating of αSyn and its dissemination in the brain. However, this notion is inconsistent with previous observations in NTG C57BL/6 J mice that intra-nigral injections of human or mouse αSyn aggregates result in identical patterns of pSer129-αSyn pathology [25]. Additionally, we found that the templated induction of pathological αSyn by mouse αSyn fibrils is an inefficient process in NTG mice compared to M20 mice. Since exogenous mouse αSyn aggregates do not necessarily recruit endogenous αSyn readily into pathological aggregates, this data suggests that the induction of αSyn pathology is a non-stochastic process dependent on the overall threshold barrier of αSyn protein. Therefore, it is possible that endogenous barriers such as a thresholding effect may regulate the induction and prionoid dissemination of αSyn pathology.

The ‘species barrier’ concept that can help explain why human WT αSyn fibrils are inefficient in seeding NTG mice is supported by the fact that there are sequence differences between human and mouse αSyn, albeit only 7 amino acids [48]. Interestingly, one of these alterations is an Ala in human αSyn compared to a Thr in mouse αSyn at residue 53, thus, mouse αSyn intrinsically has the Ala53Thr substitution that cause disease in human. In vitro mouse αSyn has a greater propensity to fibrilize into amyloid than human αSyn, akin to AlaThr53 human αSyn [49]. Although both mouse and human αSyn fibrils can cross-seed monomers of each other to polymerize into fibrils, homotypic seeding is usually more efficient in vitro [14]. Similarly, they both can initiate the aggregation of each other in vivo, but again homotypic seeding is more efficient [14]. However, as shown in extensive studies comparing the influences of amino acid alterations in mouse and human αSyn on the seeded-induction of aggregation, differences observed by in vitro cross-seeding do not always directly correlate with in vivo seeding efficiency [14]. The seeded induction of αSyn aggregation in vivo can be influenced by many differential factors unique to the αSyn seeds including cellular uptake, aggregate stability and cellular binding partners (eg. other proteins and lipids), in addition to subtle differences in the compatibility of unique conformers in initiating the recruitment and aggregation of intrinsic αSyn.

Mechanisms of de novo emergence of prions are still elusive. The aggregation of classical prions has been determined to be a non-stochastic process, whereby threshold of the prion protein determines the efficiency of prionoid transformation and propagation. Indeed, a criterion for prions is that these are formed more efficiently upon over-expression of the native protein. This was reported in the case of SUP35 and URE2 genes, where overproduction of each of these genes induce de novo formation of the respective prions [50, 51]. Indeed, in our present study as well as in previous studies, we have observed that templated induction of αSyn pathology is more efficient in mice overexpressing human αSyn than NTG mice [17, 23]. This suggests similarities between formation of classical prions and αSyn prionoid species. Overall, our study shows that the efficiency of induction of αSyn aggregation depends on the amount on available αSyn protein in the cell, suggesting that threshold of transgenic protein levels may be a determining factor for ensuring efficient prionoid induction of αSyn pathology.

In this present study and earlier studies [17], we have ascertained that M20 mice injected with αSyn aggregates show dramatic induction of astrogliosis and microgliosis. In sharp contrast to the observations by Rey et al. [26], we observed copious amounts of αSyn pathology co-localized with astrocytes, irrespective of the area of injection. This implies that, in addition to transneuronal transport, astrocytes and possibly other immune cells may also play key roles in the dissemination of pathological αSyn seeds. This has future therapeutic implications as glial cells can effectively act to reduce interneuronal transmission and more importantly clear extracellular αSyn seeds (reviewed in [52]). Indeed, it has been shown that both astrocytes and microglia can internalize and effectively degrade αSyn aggregates [53–56]. Collectively, these data suggest that glial cells that normally do not express or express very low levels of αSyn are not prone to seeding in some settings and this may play an important role in creating an endogenous barrier to the prionoid propagation of αSyn.

### Does trans-neuronal propagation of templated αSyn pathology cause DA neurodegeneration?

The etiology of DA neurodegeneration in the SN of patients with LB diseases remain unknown. One theory is that trans-neuronal transmission of seeded αSyn pathology along the neuroanatomically connected nigro-striatal pathway causes significant DA neuronal death [18, 20, 24]. However, in our study, we observed only a 20–25% DA neurodegeneration in M20 mice following injection into the IC with either mouse or human αSyn fibrils. No DA neurodegeneration was noted in NTG mice or in M20 mice injected into the CPu, an area that is directly linked to the SN. Additionally, we were intrigued by our observations that DA neurons were impervious to seeded αSyn pathology in both NTG and M20 mice. We reasoned that this observation can be attributed to multiple complex scenarios such as (1) an initial wave of pathological αSyn inclusion pathology lead to DA neurodegeneration followed by astrocytic engulfment of released materials or (2) astrocytes are able to engulf extracellular αSyn directly during the templated induction and dissemination process [38]. In an effort to localize seeded αSyn pathology in the SN, we conducted immunofluorescent co-localization studies using antibodies against non-neuronal cells and found that a majority of these inclusions are present in astrocytes, but not in the nigral TH-immunopositive DA neurons. Since we did not observe any significant nigral DA neurodegeneration in some of these mice (Cohort 3 of M20 mice), our observations suggest that astrocytes likely engulf and scavenge pathological αSyn during the propagation process. Indeed, this is consistent with observations that even direct intra-nigral injection of αSyn aggregates does not necessarily cause nigral DA neurodegeneration [20, 25] but would be contrary to observations from other groups [18, 24]. Interestingly, whether glial cells, such as astrocytes, can have a disease modifying effect in α-synucleinopathies by acting as αSyn scavengers or as unwitting vehicles that aid in dissemination of αSyn, remains to be investigated.

## Conclusions

Multiple independent reports, including this present study, show that αSyn aggregates display prionoid characteristics. Additionally, we show that contrary to expected routes of transmissibility exclusively following neuroanatomic connectivity, prionoid αSyn can be widely disseminated throughout the brain by various cellular networks - neurons as well as astrocytes. Therefore, our current findings, when aligned with the present literature, suggest that the mechanism influencing prionoid propagation of αSyn are complex and not always predictable by direct inter-neuronal connectivity. In fact, glial transmission of αSyn may also have a major impact on the pathological outcome in α-synucleinopathies. These findings are similar to disease pathogenesis by classical prion protein, where different strains of misfolded prions can have contextually-dependent transmission properties in the neuraxis [15, 16, 47]. Importantly, these properties open up potential therapeutic opportunities targeting such extracellular transfer of αSyn by utilizing endogenous immune and non-immune barriers.

## Additional file

Additional file 1: Figure S1. Transmission EM images of αSyn fibrils. Recombinant WT αSyn fibrils (human or mouse) were sonicated and analyzed by EM following uranyl acetate staining. Scale bar, 100 nm.
Additional file 2: Figure S2. Robust αSyn inclusion pathology evident in the brains of M20 mice (Cohorts 1, 2 and 3). Robust αSyn inclusion pathology was observed in areas of the brain distal from the injection site such as motor cortex, brainstem, spinal cord and SN of M20 mice injected with αSyn fibrils (A, B, C: Cohorts 1, 2 and 3 respectively). αSyn pathology was identified using pSer129-αSyn antibodies (81A and EP1536Y), Syn506 antibody and p62/Sqstm1 antibody. Both perikaryal (arrowhead) and neuritic (arrow) αSyn pathology was observed. Scale Bar, 100 μm; *n* = 3–5 mice/group.
Additional file 3: Figure S3. Robust αSyn inclusion pathology evident in the ependymal cells lining the ventricles of M20 mice (Cohorts 1 and 3). M20 mice injected with human αSyn fibrils (Cohorts 1 and 3) show robust induction of αSyn pathology in the ependymal cells lining the lateral ventricles. αSyn pathology was identified using pSer129-αSyn antibodies (81A and EP1536Y), Syn506 antibody and p62/Sqstm1 antibody. The right panel shows a magnified image of p62-immunopositive ependymal cells. None of the NTG mice injected with αSyn fibrils showed any αSyn pathology in these cells; representative images from NTG Cohort 6 is shown as an example. Hu, human; ms, mouse. *n* = 3–6/cohort; scale bar, 100 μm (all panels except right panel); 50 μm (right panel).
Additional file 4: Figure S4. αSyn pathology was not detected in TH-immunopositive DA neurons in M20 or NTG mice injected with αSyn fibrils in the striatum. Representative images showing that pSer129-αSyn inclusion pathology (red, arrows) was not localized within nigral TH-immunopositive DA neurons (green) in any of the M20 (Cohorts 2 and 3, A and B respectively) or NTG (Cohorts 4 and 6, D and E respectively) mice injected with αSyn fibrils (A-B). Few αSyn inclusions were observed in the striatal neuronal cell bodies embedded within the TH-positive terminals of M20 mice injected with human αSyn fibrils in the CPu (C). Cell nuclei were stained with DAPI (blue). The 3-color merged panel has been magnified (right panel) to visualize the localization of pSer129 αSyn inclusions in the SN. NTG mice injected with human αSyn fibrils (Cohort 5) is not shown as this cohort had no αSyn inclusion pathology. Numbers in parenthesis denote cohort number as identified in Figs. 1 and 3. Hu, human; ms, mouse. *n* = 3–6/cohort; Scale bar, 500 μm (all panels except right panel); 200 μm (merged panel, right).
Additional file 5: Figure S5. Gliosis levels in forebrain areas of line M20 mice injected with human or mouse αSyn fibrils in the striatum. Representative images of astrocytosis (GFAP immunostaining) and microgliosis (Iba-1 immunostaining) in the hippocampus and entorhinal cortex (En. Cortex) of line M20 mice injected with αSyn fibrils in the IC (Cohorts 1 and 2) or CPu (Cohort 3). Though there was an overall trend towards higher astrocytic and microglial burden in M20 mice injected with αSyn fibrils which would be co-incident with αSyn inclusion pathology present in these brain areas, there seemed to be individual variability in astroglial numbers within each group. In all cases, PBS injected mice (genotype-matched) served as controls. Different cohorts are identified by numbers in parenthesis on top of corresponding panel. Hu, human; ms, mouse. Scale bar, 100 μm; *n* = 3–5/cohort; 1 way Anova.
Additional file 6: Figure S6. Gliosis levels in forebrains of NTG mice injected with αSyn fibrils in the striatum. Representative images of astrocytosis (GFAP immunostaining) and microgliosis (Iba-1 immunostaining) in the hippocampus and entorhinal cortex (En. Cortex) of NTG mice injected with WT αSyn fibrils in the IC (Cohorts 4 and 5) or CPu (Cohort 6). No significant alterations in GFAP staining was observed in any of the cohorts. Increased microgliosis was observed in the entorhinal cortex of NTG mice injected with mouse αSyn fibrils in the IC (**p* < 0.05) but not in the hippocampus of these mice. Strikingly, dampening of microgliosis was observed in forebrain areas of mice injected with human αSyn (Cohort 5; hippocampus, **p* < 0.05; entorhinal cortex, *p* > 0.05) as well as in the hippocampus and cortex of NTG mice injected with mouse αSyn fibrils (Cohort 6, ***p* < 0.01). In all cases, PBS injected mice (genotype-matched) served as controls. Different cohorts are identified by numbers in parenthesis on top of panel. Hu, human; ms, mouse. Scale bar, 100 μm; *n* = 3–6/cohort; 1 way Anova, **p* < 0.05, ***p* < 0.01.
Additional file 7: Figure S7. Astrocytic αSyn inclusions were abundant in midbrain and forebrain areas of line M20 mice injected with human αSyn fibrils (Cohorts 1 and 3). Representative immunofluorescence staining showing co-localization of pSer129 immunoreactive αSyn pathology (red) within the GFAP immunopositive astrocytes (green) in the ventral midbrain, hippocampus and motor cortex of human αSyn fibril injected M20 mice. In both cohorts (1 and 3) of these M20 mice, astrocytes laden with pSer129-αSyn were present in abundance (arrows). Additionally, several LB type inclusions or neurites (arrowheads) were also present in cells that were not immunopositive for GFAP. Cell nuclei were stained with DAPI (blue). The 3-color merged panel has been magnified (right panel) to visualize the localization of astrocytic pSer129 αSyn. Hu, human; ms, mouse. *n* = 3–5/cohort; Scale bar, 500 μm (all panels except right panel); 200 μm (merged panel, right).
Additional file 8: Figure S8. Astrocytic αSyn inclusions were absent in midbrain and forebrain areas of NTG mice injected with mouse αSyn fibrils (Cohorts 4 and 6). Representative immunofluorescence staining showing that pSer129 immunoreactive αSyn pathology (red; A-B) does not colocalize within the GFAP immunopositive astrocytes (green; A-B) in the ventral midbrain, hippocampus and motor cortex of mouse αSyn fibril injected NTG mice (Cohorts 4 and 6). Of note, most of these mice have low numbers of pSer129-αSyn pathology overall. Cell nuclei were stained with DAPI (blue). The 3-color merged panel has been magnified (right panel) to visualize the localization of pSer129 αSyn. Hu, human; ms, mouse. *n* = 3–6/cohort; Scale bar, 500 μm (all panels except right panel); 200 μm (merged panel, right). (PDF 17097 kb)

## Abbreviations

CPu: caudate putamen
DA: dopaminergic
EM: electron microscopy
GFAP: glial fibrillary acidic protein
Iba-1: ionized calcium-binding adapter molecule 1
IC: internal capsule
LB: Lewy body
NTG: nontransgenic
pSer129: αSyn phosphorylated on Serine 129
SN: substantia nigra
Sqstm1: Sequestosome1
TH: Tyrosine Hydroxylase
αSyn: α-synuclein
