# Supplementary figures and images for: Intrastriatal injection of α-synuclein can lead to widespread synucleinopathy independent of neuroanatomic connectivity
Source: Mol Neurodegener. 2017 May 29;12:40. doi: 10.1186/s13024-017-0182-z (PMC5447308; doi:10.1186/s13024-017-0182-z)

Human WT  $\alpha$ -syn fibril

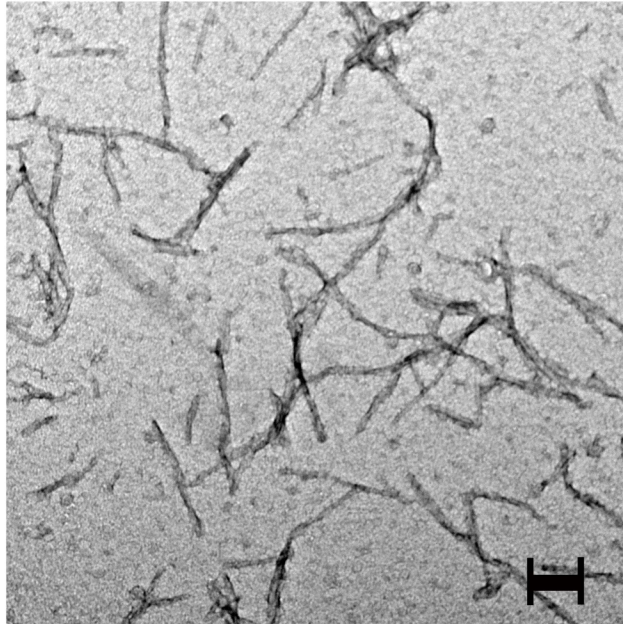

Mouse WT  $\alpha$ -syn fibril

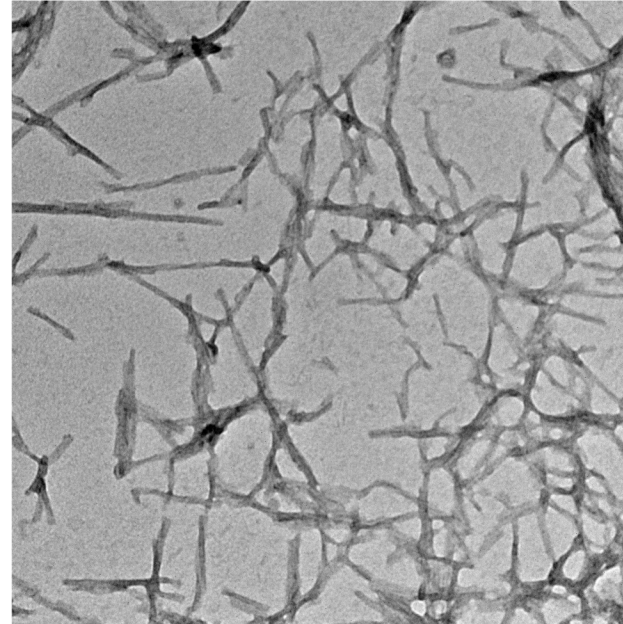

Figure S1

Supplement: Additional file 1: Figure S1. — Transmission EM images of αSyn fibrils. Recombinant WT αSyn fibrils (human or mouse) were sonicated and analyzed by EM following uranyl acetate staining. Scale bar, 100 nm. [file 13024_2017_182_MOESM1_ESM.pdf]

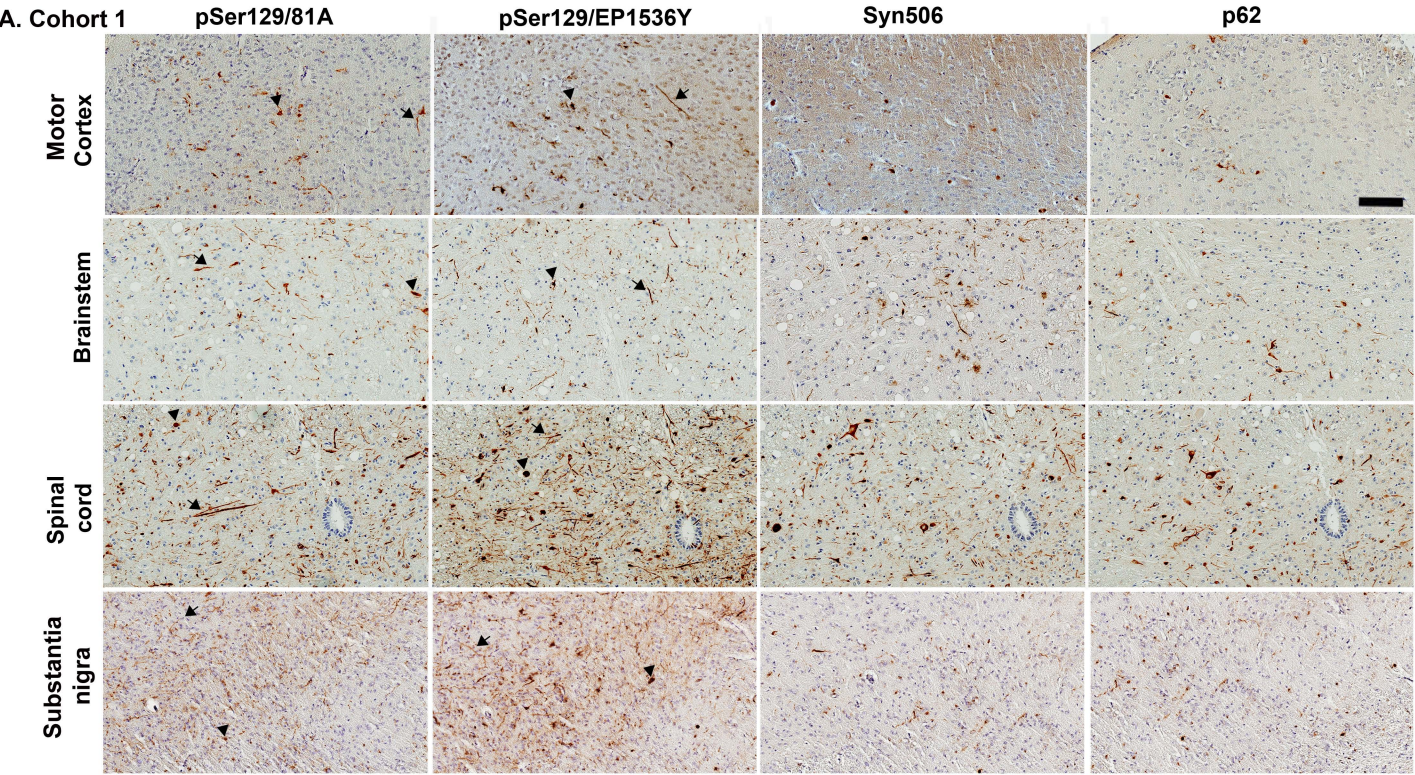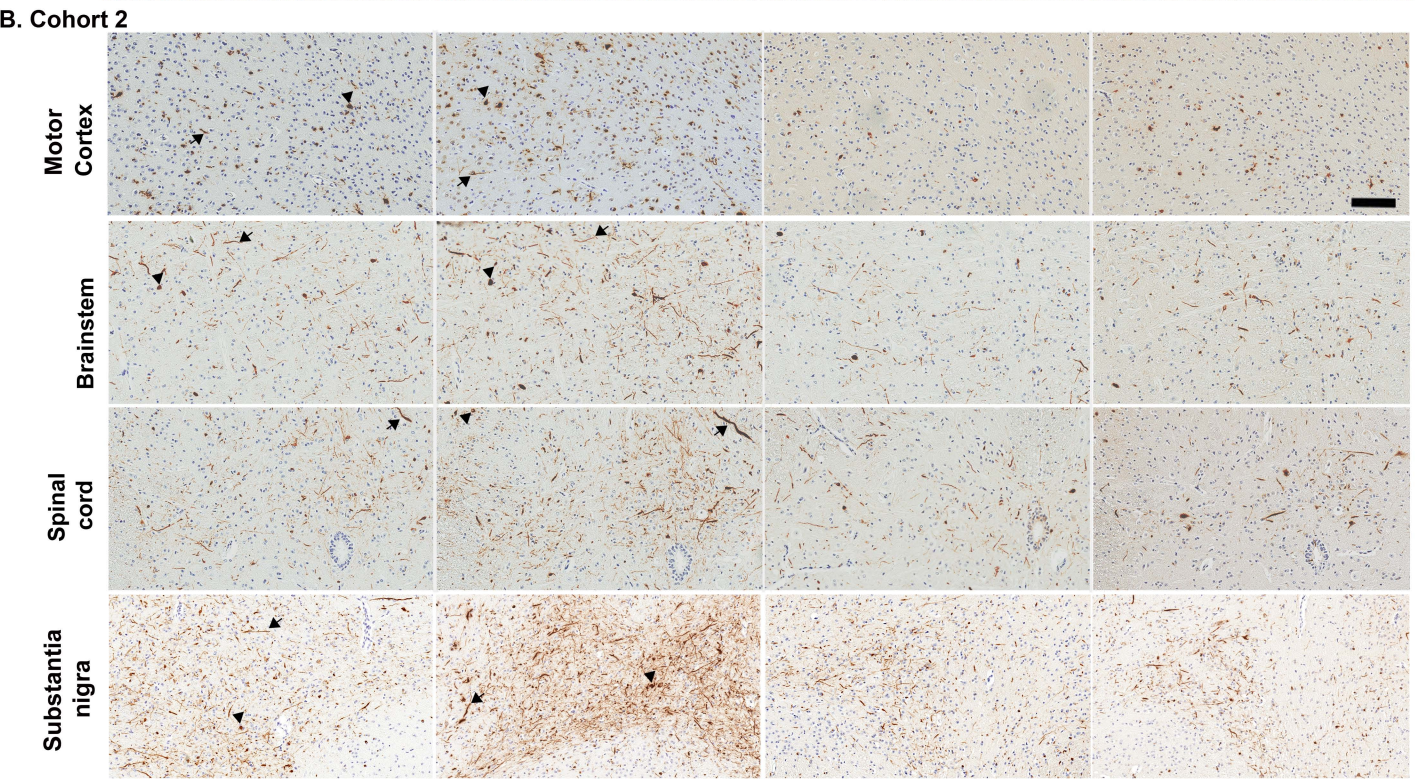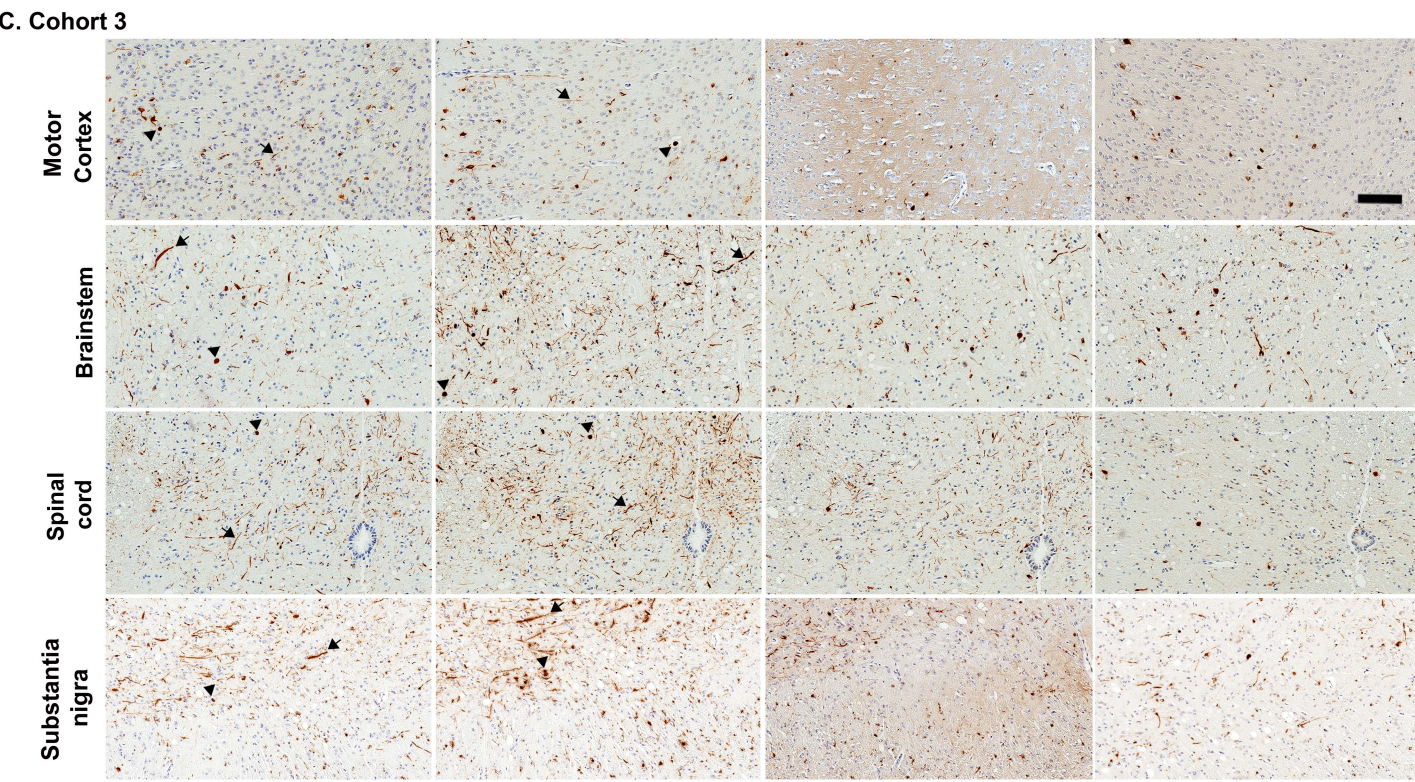

Figure S2

Supplement: Additional file 2: Figure S2. — Robust αSyn inclusion pathology evident in the brains of M20 mice (Cohorts 1, 2 and 3). Robust αSyn inclusion pathology was observed in areas of the brain distal from the injection site such as motor cortex, brainstem, spinal cord and SN of M20 mice injected with αSyn fibrils (A, B, C: Cohorts 1, 2 and 3 respectively). αSyn pathology was identified using pSer129-αSyn antibodies (81A and EP1536Y), Syn506 antibody and p62/Sqstm1 antibody. Both perikaryal (arrowhead) and neuritic (arrow) αSyn pathology was observed. Scale Bar, 100 μm; n = 3–5 mice/group. [file 13024_2017_182_MOESM2_ESM.pdf]

NTG:ms  $\alpha$ Syn cohort 6 M20:hu  $\alpha$ Syn cohort 3 M20:hu  $\alpha$ Syn cohort 1

pSer129/81A

pSer129/EP1536Y

Syn506

p62

p62

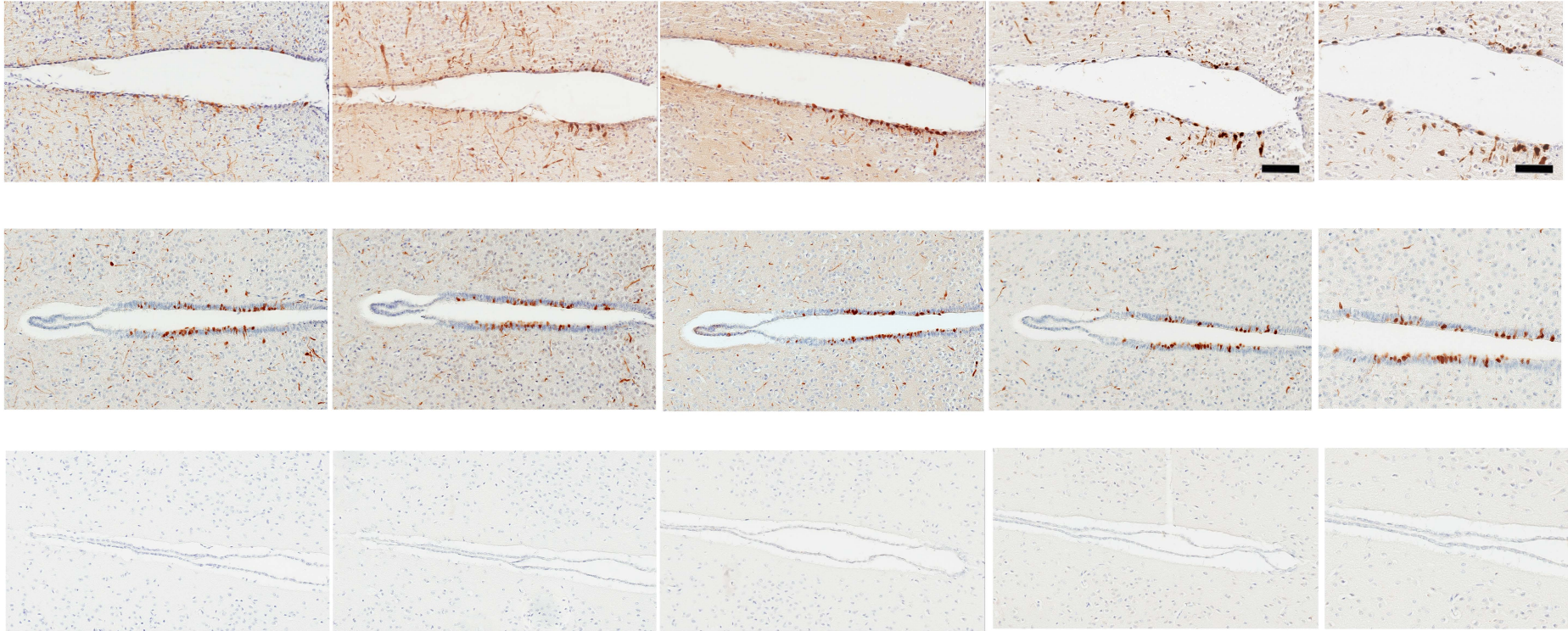

Figure S3

Supplement: Additional file 3: Figure S3. — Robust αSyn inclusion pathology evident in the ependymal cells lining the ventricles of M20 mice (Cohorts 1 and 3). M20 mice injected with human αSyn fibrils (Cohorts 1 and 3) show robust induction of αSyn pathology in the ependymal cells lining the lateral ventricles. αSyn pathology was identified using pSer129-αSyn antibodies (81A and EP1536Y), Syn506 antibody and p62/Sqstm1 antibody. The right panel shows a magnified image of p62-immunopositive ependymal cells. None of the NTG mice injected with αSyn fibrils showed any αSyn pathology in these cells; representative images from NTG Cohort 6 is shown as an example. Hu, human; ms, mouse. n = 3–6/cohort; scale bar, 100 μm (all panels except right panel); 50 μm (right panel). [file 13024_2017_182_MOESM3_ESM.pdf]

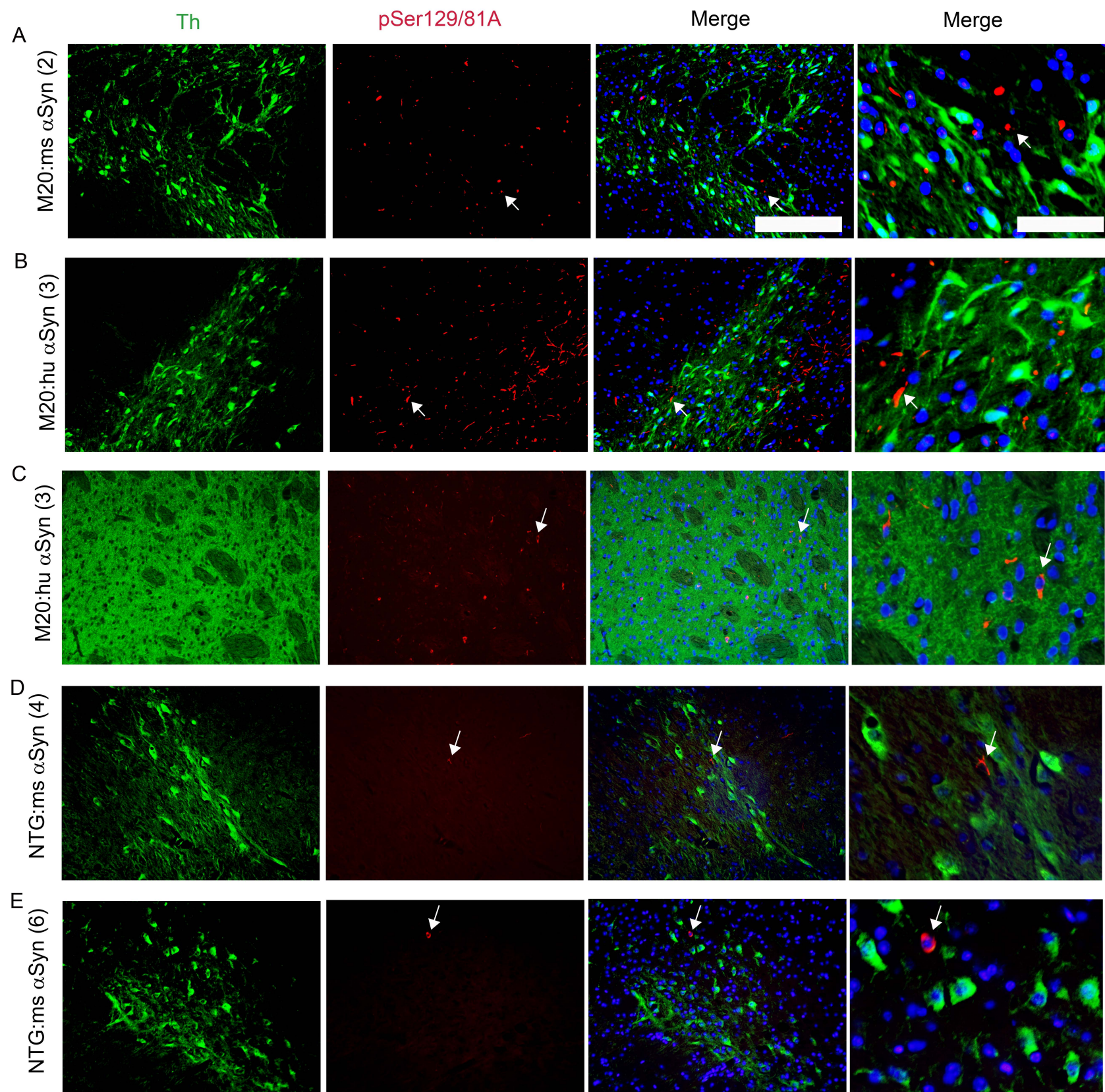

Figure S4

Supplement: Additional file 4: Figure S4. — αSyn pathology was not detected in TH-immunopositive DA neurons in M20 or NTG mice injected with αSyn fibrils in the striatum. Representative images showing that pSer129-αSyn inclusion pathology (red, arrows) was not localized within nigral TH-immunopositive DA neurons (green) in any of the M20 (Cohorts 2 and 3, A and B respectively) or NTG (Cohorts 4 and 6, D and E respectively) mice injected with αSyn fibrils (A-B). Few αSyn inclusions were observed in the striatal neuronal cell bodies embedded within the TH-positive terminals of M20 mice injected with human αSyn fibrils in the CPu (C). Cell nuclei were stained with DAPI (blue). The 3-color merged panel has been magnified (right panel) to visualize the localization of pSer129 αSyn inclusions in the SN. NTG mice injected with human αSyn fibrils (Cohort 5) is not shown as this cohort had no αSyn inclusion pathology. Numbers in parenthesis denote cohort number as identified in Figs. 1 and 3. Hu, human; ms, mouse. n = 3–6/cohort; Scale bar, 500 μm (all panels except right panel); 200 μm (merged panel, right). [file 13024_2017_182_MOESM4_ESM.pdf]

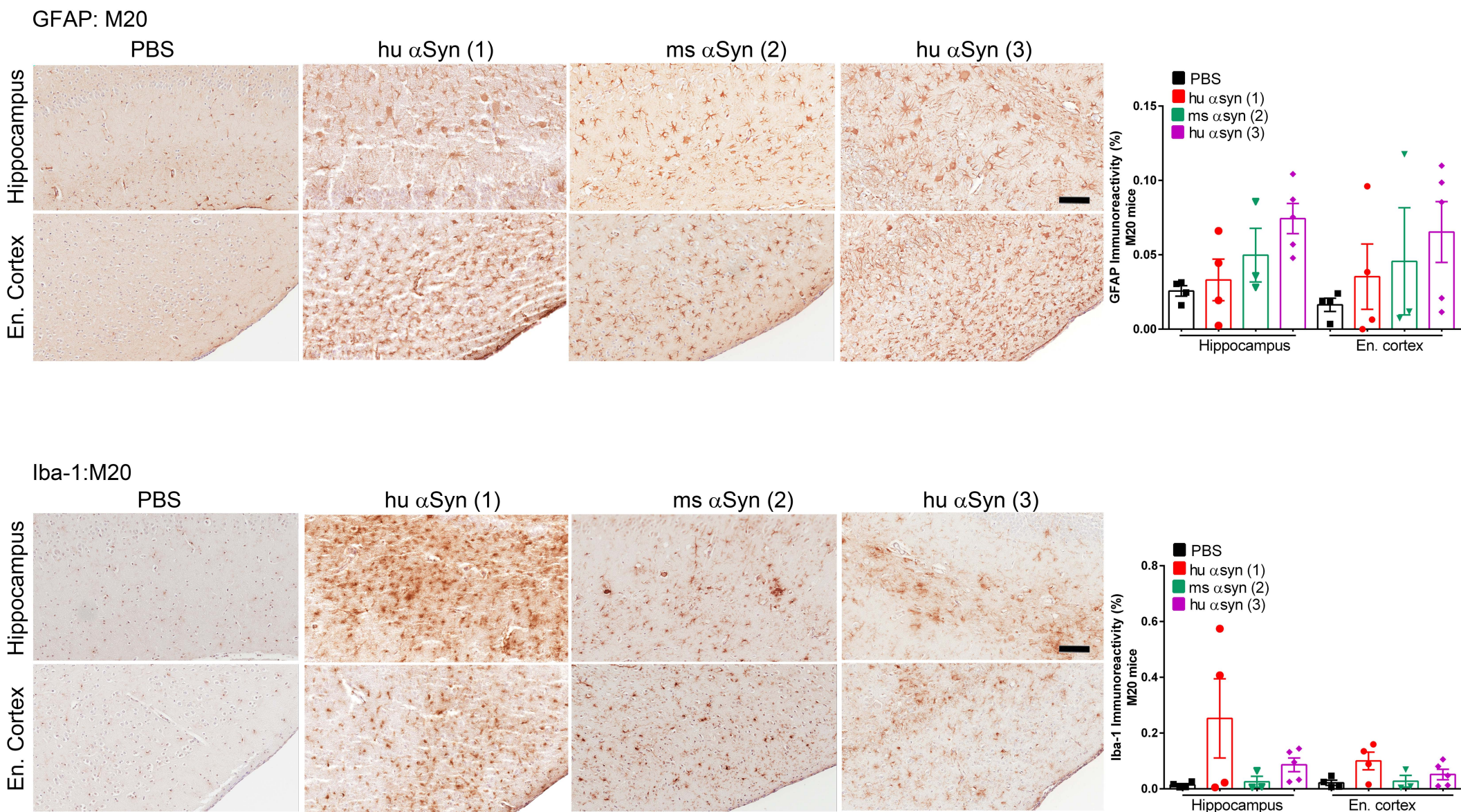

Figure S5

Supplement: Additional file 5: Figure S5. — Gliosis levels in forebrain areas of line M20 mice injected with human or mouse αSyn fibrils in the striatum. Representative images of astrocytosis (GFAP immunostaining) and microgliosis (Iba-1 immunostaining) in the hippocampus and entorhinal cortex (En. Cortex) of line M20 mice injected with αSyn fibrils in the IC (Cohorts 1 and 2) or CPu (Cohort 3). Though there was an overall trend towards higher astrocytic and microglial burden in M20 mice injected with αSyn fibrils which would be co-incident with αSyn inclusion pathology present in these brain areas, there seemed to be individual variability in astroglial numbers within each group. In all cases, PBS injected mice (genotype-matched) served as controls. Different cohorts are identified by numbers in parenthesis on top of corresponding panel. Hu, human; ms, mouse. Scale bar, 100 μm; n = 3–5/cohort; 1 way Anova. [file 13024_2017_182_MOESM5_ESM.pdf]

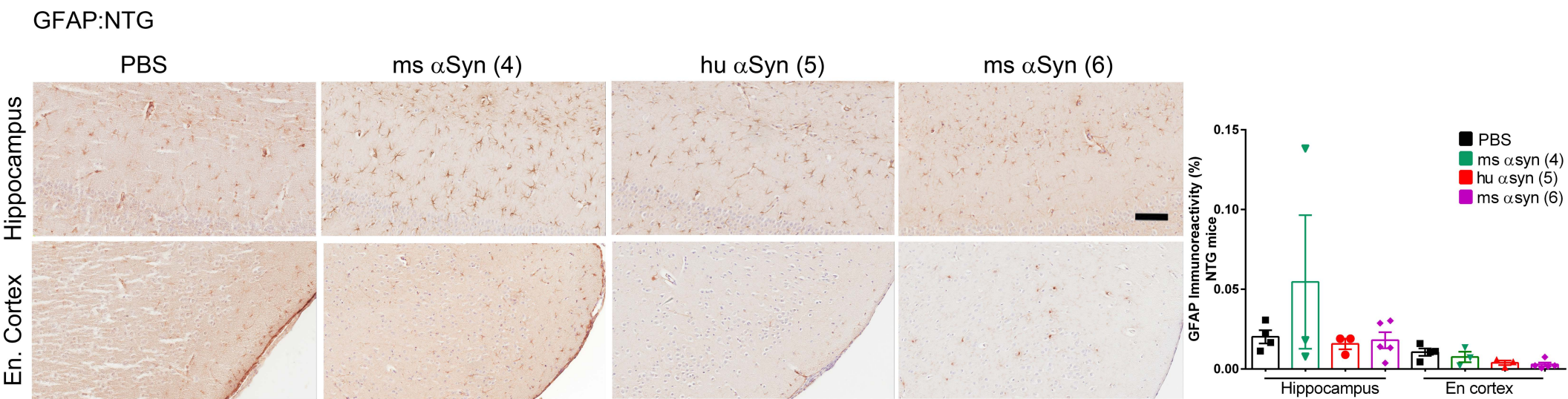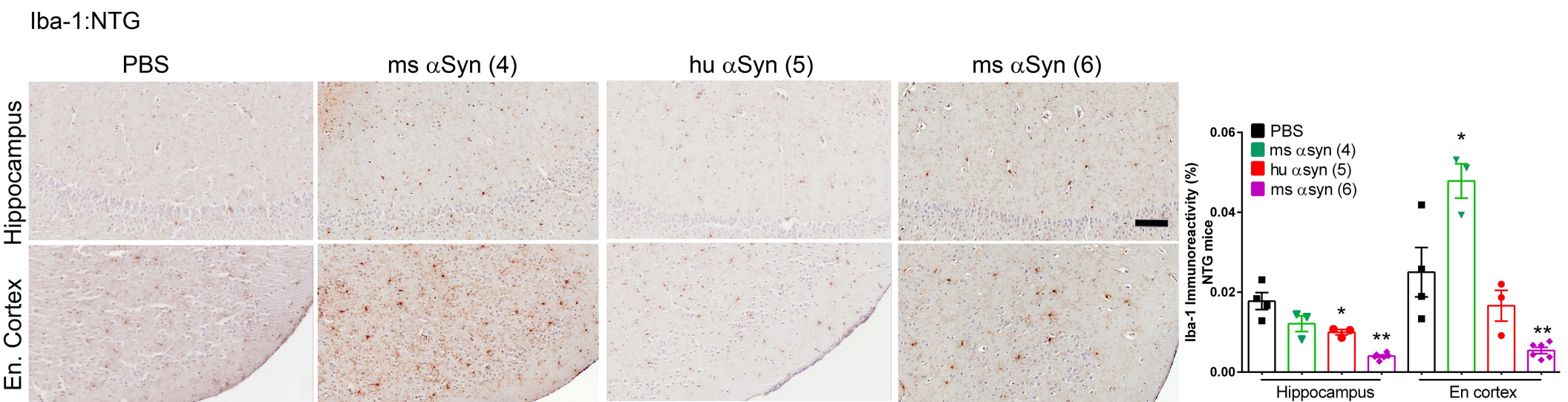

Figure S6

Supplement: Additional file 6: Figure S6. — Gliosis levels in forebrains of NTG mice injected with αSyn fibrils in the striatum. Representative images of astrocytosis (GFAP immunostaining) and microgliosis (Iba-1 immunostaining) in the hippocampus and entorhinal cortex (En. Cortex) of NTG mice injected with WT αSyn fibrils in the IC (Cohorts 4 and 5) or CPu (Cohort 6). No significant alterations in GFAP staining was observed in any of the cohorts. Increased microgliosis was observed in the entorhinal cortex of NTG mice injected with mouse αSyn fibrils in the IC (*p < 0.05) but not in the hippocampus of these mice. Strikingly, dampening of microgliosis was observed in forebrain areas of mice injected with human αSyn (Cohort 5; hippocampus, *p < 0.05; entorhinal cortex, p > 0.05) as well as in the hippocampus and cortex of NTG mice injected with mouse αSyn fibrils (Cohort 6, **p < 0.01). In all cases, PBS injected mice (genotype-matched) served as controls. Different cohorts are identified by numbers in parenthesis on top of panel. Hu, human; ms, mouse. Scale bar, 100 μm; n = 3–6/cohort; 1 way Anova, *p < 0.05, **p < 0.01. [file 13024_2017_182_MOESM6_ESM.pdf]

A. M20 hu  $\alpha$ syn cohort 1

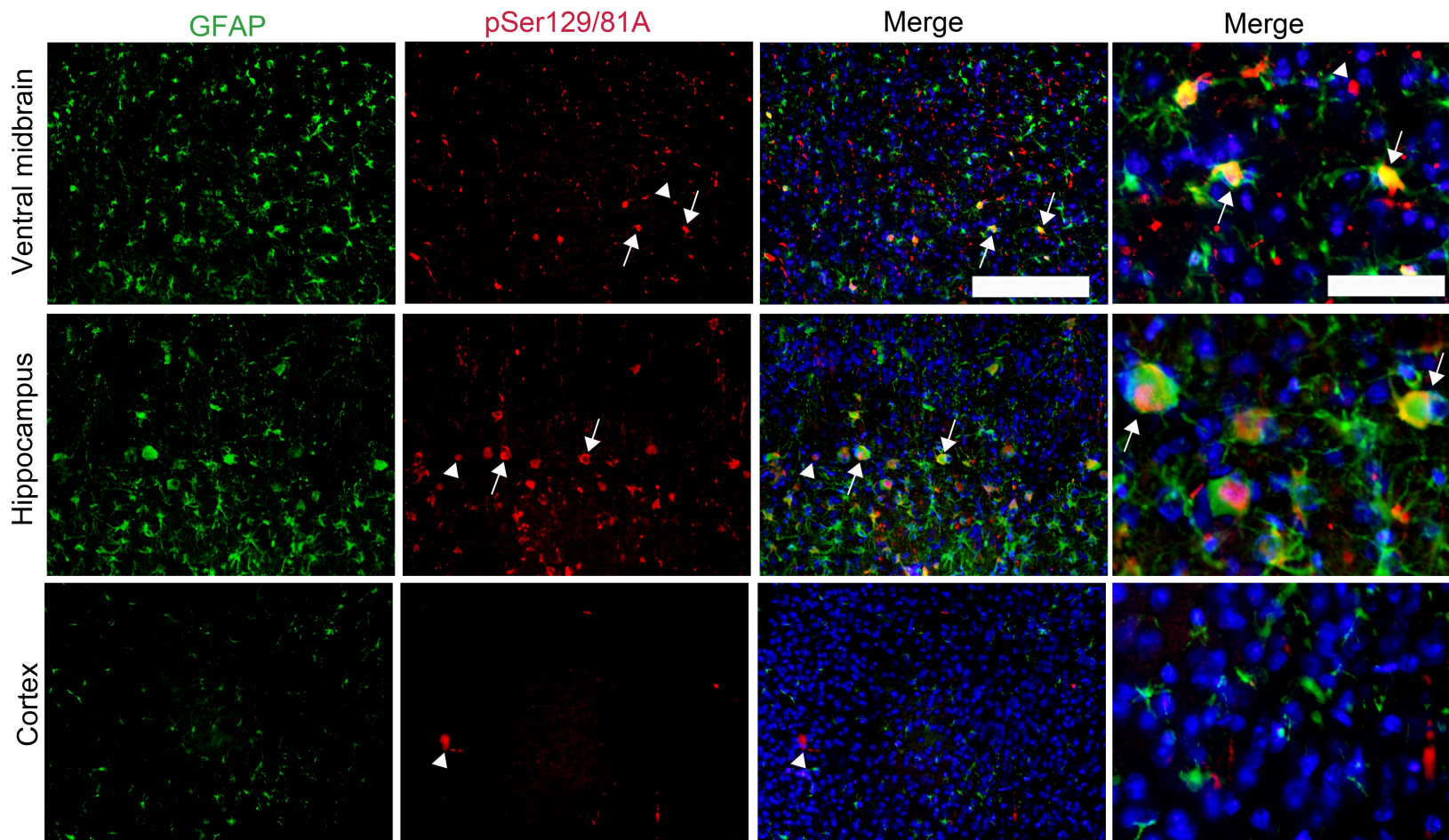

B. M20 hu  $\alpha$ syn cohort 3

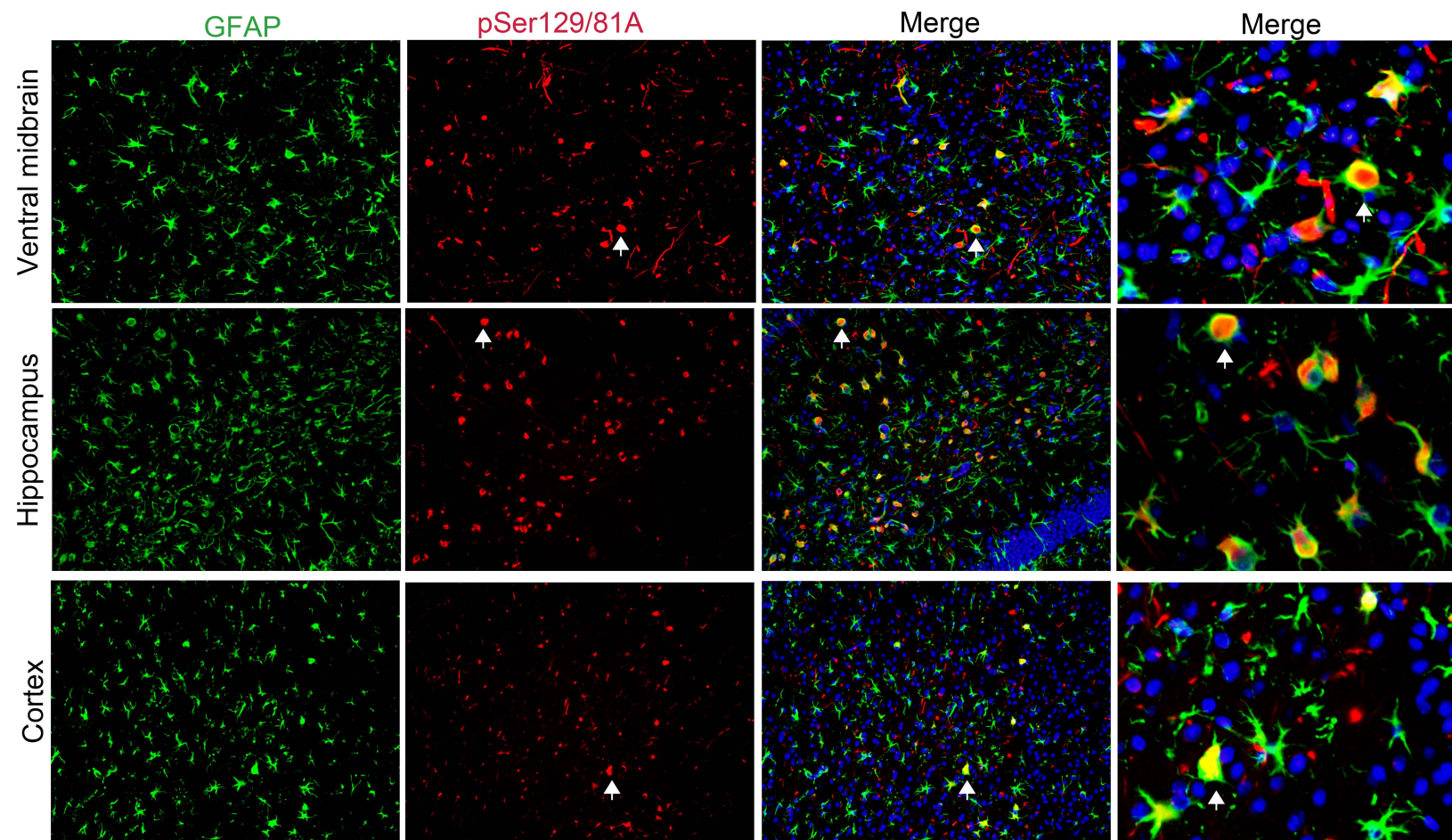

Figure S7

Supplement: Additional file 7: Figure S7. — Astrocytic αSyn inclusions were abundant in midbrain and forebrain areas of line M20 mice injected with human αSyn fibrils (Cohorts 1 and 3). Representative immunofluorescence staining showing co-localization of pSer129 immunoreactive αSyn pathology (red) within the GFAP immunopositive astrocytes (green) in the ventral midbrain, hippocampus and motor cortex of human αSyn fibril injected M20 mice. In both cohorts (1 and 3) of these M20 mice, astrocytes laden with pSer129-αSyn were present in abundance (arrows). Additionally, several LB type inclusions or neurites (arrowheads) were also present in cells that were not immunopositive for GFAP. Cell nuclei were stained with DAPI (blue). The 3-color merged panel has been magnified (right panel) to visualize the localization of astrocytic pSer129 αSyn. Hu, human; ms, mouse. n = 3–5/cohort; Scale bar, 500 μm (all panels except right panel); 200 μm (merged panel, right). [file 13024_2017_182_MOESM7_ESM.pdf]

A. NTG ms  $\alpha$ syn Cohort 4

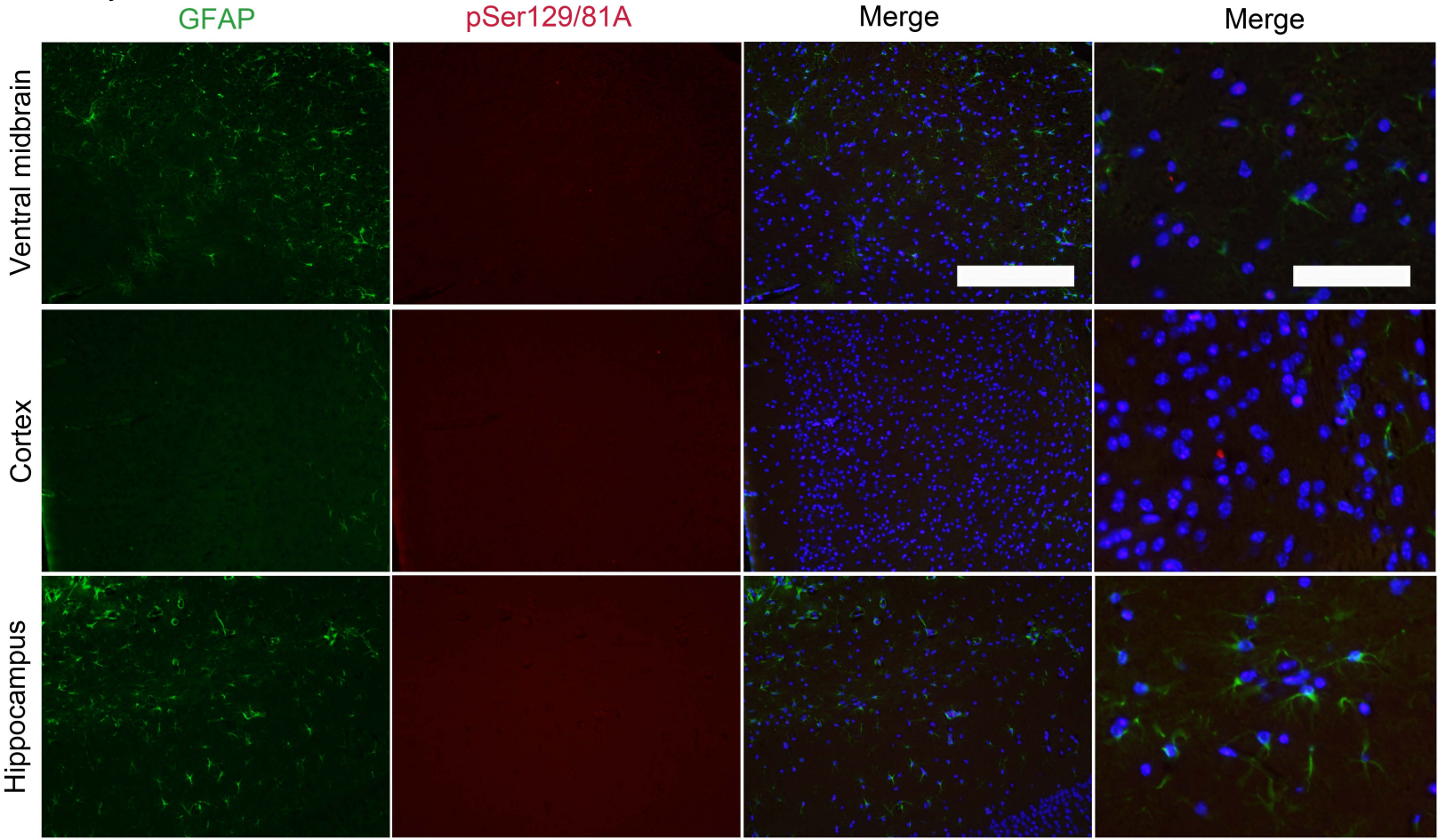

B. NTG ms  $\alpha$ syn Cohort 6

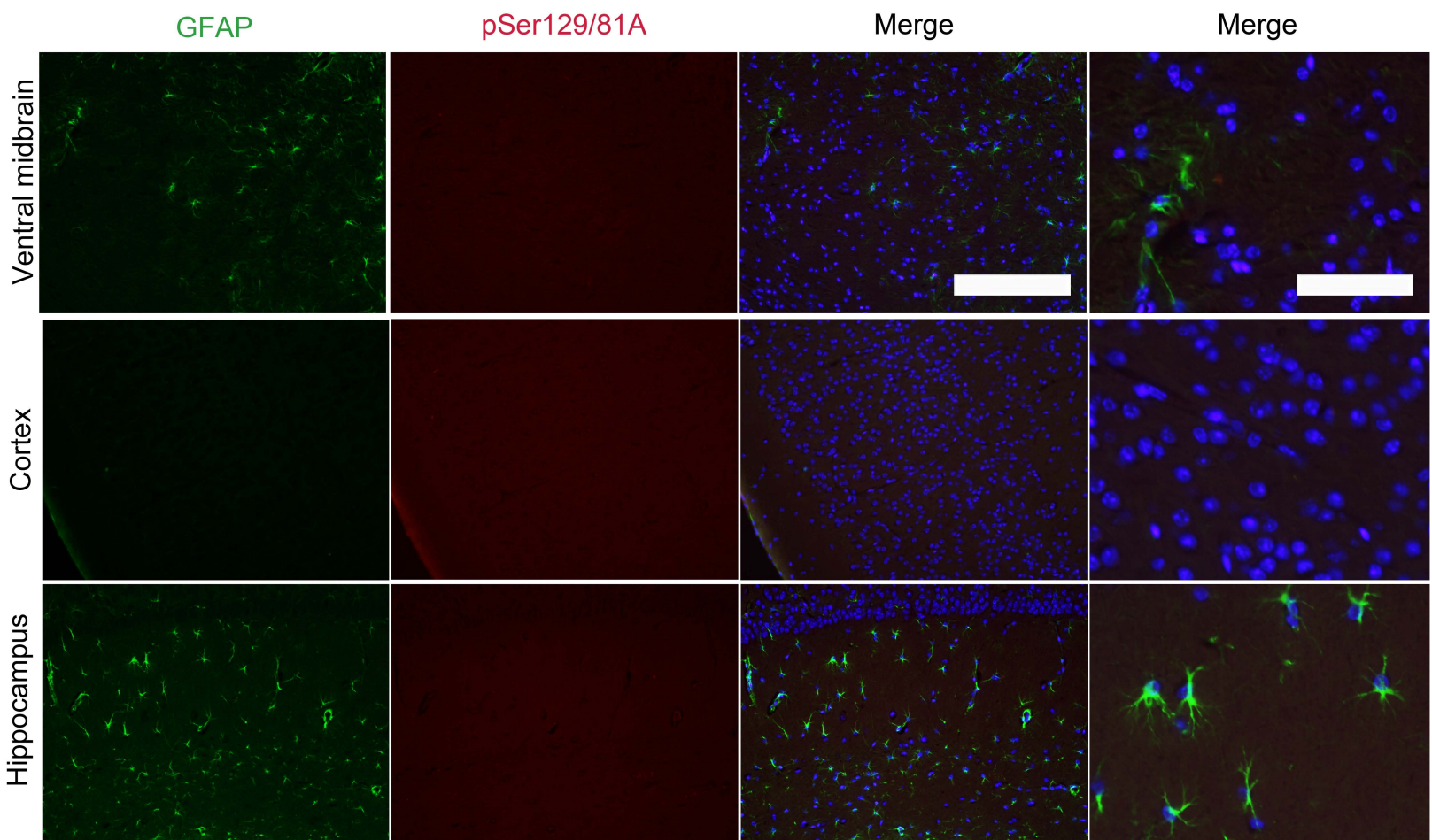

Figure S8

Supplement: Additional file 8: Figure S8. — Astrocytic αSyn inclusions were absent in midbrain and forebrain areas of NTG mice injected with mouse αSyn fibrils (Cohorts 4 and 6). Representative immunofluorescence staining showing that pSer129 immunoreactive αSyn pathology (red; A-B) does not colocalize within the GFAP immunopositive astrocytes (green; A-B) in the ventral midbrain, hippocampus and motor cortex of mouse αSyn fibril injected NTG mice (Cohorts 4 and 6). Of note, most of these mice have low numbers of pSer129-αSyn pathology overall. Cell nuclei were stained with DAPI (blue). The 3-color merged panel has been magnified (right panel) to visualize the localization of pSer129 αSyn. Hu, human; ms, mouse. n = 3–6/cohort; Scale bar, 500 μm (all panels except right panel); 200 μm (merged panel, right). (PDF 17097 kb) [file 13024_2017_182_MOESM8_ESM.pdf]
